# Poaching of protected wolves fluctuated seasonally and with non-wolf hunting

**DOI:** 10.1038/s41598-022-05679-w

**Published:** 2022-02-02

**Authors:** Francisco J. Santiago-Ávila, Adrian Treves

**Affiliations:** Nelson Institute for Environmental Studies, University of Wisconsin – Madison, Madison, USA

**Keywords:** Population dynamics, Conservation biology

## Abstract

Poaching is the main cause of mortality for many large carnivores, and mitigating it is imperative for the persistence of their populations. For Wisconsin gray wolves (*Canis lupus*), periods of increased risk in overall mortality and poaching seem to overlap temporally with legal hunting seasons for other large mammals (hunting wolves was prohibited). We analyzed monitoring data from adult, collared wolves in Wisconsin, USA (1979–2012, n = 495) using a competing-risk approach to test explicitly if seasons during which it was legal to train hunting hounds (hounding) or hunt other large mammals (hunting) affected wolves’ hazard of cause-specific mortality and disappearance. We found increases in hazard for disappearances and documented (‘reported’) poaching during seasons with hunting, hounding or snow cover relative to a season without these factors. The ‘reported poached’ hazard increased > 650% during seasons with hunting and snow cover, which may be due to a seasonal surge in numbers of potential poachers or to some poachers augmenting their activities. Snow cover was a major environmental factor contributing to poaching, presumably through increased detection of wolves. Our study suggests poaching is by far the highest mortality hazard for wolves and reinforces the need for protections and policies targeting poaching of protected populations.

## Introduction

Humans threaten the survival of large carnivores and the viability of their populations through habitat loss, killing and prey depletion^1^. Consequently, the contraction, depletion and extirpation of large carnivores has contributed to simplification of trophic structures linked to both lower biodiversity and degraded ecosystem functions^1–3^, suggesting the elimination of large carnivores “is one of the most significant anthropogenic impacts on nature”^1,3^. Moreover, there is a growing concern for the wellbeing and claims of individual nonhuman animals and large carnivores within conservation^4–6^. Increased consideration of nonhuman claims demands robust assessments of how anthropogenic activities, including those aimed at other species, impact risk of harm, including death^7,8^.

Importantly, poaching, both reported and cryptic, is the main form of anthropogenic mortality for various regions’ carnivores^9–14^; including four US wolf (*Canis lupus*, *Canis rufus, Canis lupus baileyi*) populations^15–18^. Here, we distinguish between these two poaching variants by their detection on the landscape, following^12,15,17,18^: while ‘reported poaching’ refers to the component of total poaching that is reported, evidenced and thus detected by management agencies, ‘cryptic poaching’ refers to poaching that remains concealed and thus undetected. The concealment of poaching (its cryptic component) contributes to its systematic underestimation^12,15,17–19^, increasing concerns over the viability of large carnivore populations subject to additional sources of anthropogenic mortality^20–23^. Given both its prevalence and cryptic nature, mitigating poaching seems imperative for the persistence of many large carnivore populations, including endangered ones that are not subject to hunting seasons^10,11,16–18,24^.

For wolf populations in the US, recent research has explored the effect of reducing protections for the species on cause-specific mortality, including poaching and its cryptic variant. Invariably, such studies have found an increase in poaching risk or incidence during policy time periods when species protections are reduced; i.e., when targeted lethal management by agency personnel, rather than unselective public hunting seasons, is sanctioned^17,18^. For Wisconsin wolves, results are largely consistent with research detecting unmeasured mortality necessary to account for the slowdown in population growth during periods of reduced protections in that population^25–27^. Relative to full protection periods, wolves in Minnesota also face an increased risk of overall anthropogenic mortality and poaching once protections are reduced, including public hunting, even if protections are later reinstated^28^.

Research on intra-year mortality risk for Wisconsin wolves also found that periods of increased risk in overall mortality and poaching overlapped with hunting seasons for other large mammals, such as white-tailed deer (*Odocoileus virginianus*) and black bear (*Ursus americanus*), and hypothesized such increases in poaching risk were in part attributable to the surge of hunters on the landscape during those periods^14,18,29^. Similarly, in Minnesota, Nov–Apr is the period of highest overall anthropogenic and illegal killing of wolves, with the authors again pointing to the overlap with firearm season for white-tailed deer^28^. Critically endangered red wolves in the Southeastern US also face increased risk of anthropogenic mortality (mostly attributable to gunshot) and disappearances during fall and winter hunting seasons for other large mammals^16,24^.

The estimated increases in anthropogenic and illegal killing of wolves during other large mammal hunting seasons is also supported by social science research on hunter motivations and inclinations to poach wolves. Various surveys of Wisconsin residents spanning over a decade, and two qualitative focus groups, revealed rising inclinations to poach after federal protections were reduced and the state sanctioned lethal management^30–32^. Treves et al.^30^ found that increased inclination to poach wolves was correlated with perception of competition over deer, rather than fear or loss of domestic animals. Moreover, a quarter of bear hunters in that study said they would poach a wolf. Subsequent focus group research revealed that bear hunters generally hold negative attitudes towards wolves and wolf management, and that they “…believe that bear hunters, in general, sanction the illegal killing of wolves”^31^, p. 6. Farmers’ attitudes toward wolves did not differ significantly from those of hunters, and they believed that most farmers “approved, or were at least tolerant, of illegally killing wolves” (p. 6) The same study revealed deer hunters hold a range of attitudes towards wolves, significantly more positive than farmers or bear hunters, yet with some endorsement or participation in their illegal killing. Later survey research by Hogberg et al.^32^ highlighted a continuing negative trend in attitudes among male respondents and hunters living in wolf range before and after the state’s first legal hunt in 2012. All studies found net shifts towards agreement with the perception that wolves threaten deer hunting opportunities.

In this study, we analyze monitoring data from adult, collared wolves in Wisconsin, USA (1979–2012, n = 495 collared adults) to test explicitly if seasons during which it was legal to train hunting hounds (hounding) or hunt other large mammals (hunting wolves was prohibited; see “Methods” section) affected wolves’ hazard of cause-specific mortality and disappearance (endpoints hereafter). Our explicit modelling of intra-wolf-year anthropogenic and natural seasons allows us to explore any interactions between endpoints within seasons, as well as interactions between anthropogenic and natural landscape conditions (e.g., simultaneous hunting and snow cover). Our results suggest poaching hazard, both cryptic and reported, is substantially higher during seasons with hunting and snow cover relative to seasons without these factors. Our methods can promote the conservation and consideration of wild animals through improving the evaluation of anthropogenic impacts on their mortality and disappearances, as well as the effectiveness of policies aimed at protecting them and mitigating poaching.

## Results

### Estimating unconditional, endpoint-specific hazards

We built 3 stratified (by endpoint and protection period [lib_kill]), joint Cox models (see model statistics in Supplementary Material Table 3). We present results by endpoint for our best model (Table 1), following our model selection criteria (see Supplementary Material Tables 4–6 for results from Models 1 and 2). Results, largely consistent across models (Table 1, Supplementary Material Tables 4–6; and see Supplementary Material Table 7 for analogous ‘known-LTF’ model), reveal that both anthropogenic and natural seasons were associated with meaningful increases in the hazard of multiple endpoints for collared adult wolves, especially of reported poached.

**Table 1:** Hazard ratio (HR) point estimates from the stratified (by endpoint and protection period) joint Cox Model 3 (our best performing model, see Supplementary Material Tables 3–7 for model statistics, diagnostics and other models) for n = 495 monitored adult wolves, by endpoint and season (LTF = ‘lost to follow-up’, defined in Methods).

Season	Hunt/hound			Hunt/hound/snow			Snow		
Endpoint	HR (se)	95 CI		HR (se)	95 CI		HR (se)	95 CI	
LTF	1.18	0.72	1.95	1.19	0.65	2.19	1.52	0.90	2.55
	(− 0.30)			(− 0.37)			(− 0.40)		
Legal	1.78	0.79	4.05	0.72	0.11	4.71	0.00	0.00	0.00
	(− 0.75)			(− 0.69)			(.)		
Reported poached	1.23	0.52	2.91	7.58***	3.19	17.99	3.27***	1.36	7.86
	(− 0.54)			(− 3.34)			(− 1.46)		
Natural	238.98***	8.61	6630.66	392.14***	11.29	13,614.75	623.97***	7.00	55,655.22
	(− 405.19)			(− 709.72)			(− 1429.70)		
Unknown	1.20	0.33	4.44	7671.73***	15.11	3,894,537.72	0.66	0.13	3.32
	(− 0.80)			(− 24,384.75)			(− 0.54)		
Collision	2.61	0.49	14.06	0.23	0.02	3.00	1.17	0.19	7.27
	(− 2.24)			(− 0.30)			(− 1.09)		
**tvc − (ln(t))**									
Natural	0.39***	0.23	0.66	0.44***	0.25	0.77	0.41**	0.20	0.82
	(− 0.11)			(− 0.13)			(− 0.14)		
Unknown	–	–	–	0.17***	0.05	0.55	–	–	–
	–			(− 0.10)			–		

We present HRs and compatibility intervals (95 CI) for all endpoint-season interactions relative to a baseline season.

*p < 0.10, **p < 0.05, ***p < 0.01.

#### Lost-to-follow-up (LTF)

Hounding and hunting seasons without snow (hunt/hound; Jul–Nov 14th) were associated with an 18% (HR 1.18, 95 CI 0.72–1.95) increase in hazard of LTF relative to the baseline period (April 15th–June). Similarly, the hunting and snow season (hunt/hound/snow) increased the hazard of LTF by 19% (HR 1.19, 95 CI 0.65–2.19). The snow season outside hunting or hounding periods (snow) increased the relative hazard of a wolf going LTF by 52% (HR 1.52, 95 CI 0.9–2.55).

#### Legal

Snowless hounding and hunting seasons (hunt/hound) were associated with a 78% increase in hazard of legal killing for wolves (HR 1.78, 95 CI 0.72–1.95), relative to the baseline season. On the other hand, hounding and hunting seasons with snow (hunt/hound/snow) decreased the hazard of a wolf being killed legally by 28% (HR 0.72, 95 CI 0.11–4.71). There were no records of wolves being killed legally during the snow season.

#### Reported poached

The hunt/hound season increased the hazard of wolves being reported poached by 23% (HR 1.23, 95 CI 0.79–4.05). The hunt/hound/snow period was associated with the highest hazard of wolves being reported poached, with a substantial increase of 658% over the baseline season (HR 7.58, 3.19–17.99). The snow season without hounding or hunting (snow) was associated with another substantial, albeit lower than with hunting, increase in hazard for wolves being poached and reported, this time by 227% (HR 3.27, 95 CI 1.36–7.86).

#### Natural, unknown and collision

The hazard of a natural endpoint showed substantial initial increases in hazard relative to baseline for all seasons, but with considerable non-proportional decreases in hazard with monitoring time (Table 1). The natural endpoint saw higher increases in hazard during the snow (HR 623.97, tvc = 0.41) and hunt/hound/snow (HR 392.14, tvc = 0.44) seasons than for the snowless hunt/hound season (HR 238.98, tvc = 0.39). The hazard of an unknown endpoint increased during hunt/hound (HR 1.2), increased during hunt/hound/snow seasons but with a considerable non-proportional decrease over time (HR 7671.73, tvc = 0.17), and decreased during the snow season (HR 0.66). The hazard of wolves dying by collisions increased during the hunt/hound (HR 2.61) and snow seasons (HR 1.17), and decreased during the hunt/hound/season (HR 0.23). The low number of events per variable (EPV, see Methods) for both the unknown and collision endpoints reduce our confidence in their results.

### Analysis of cumulative hazards and incidences over monitoring time 't', by season

**Figure 1:**
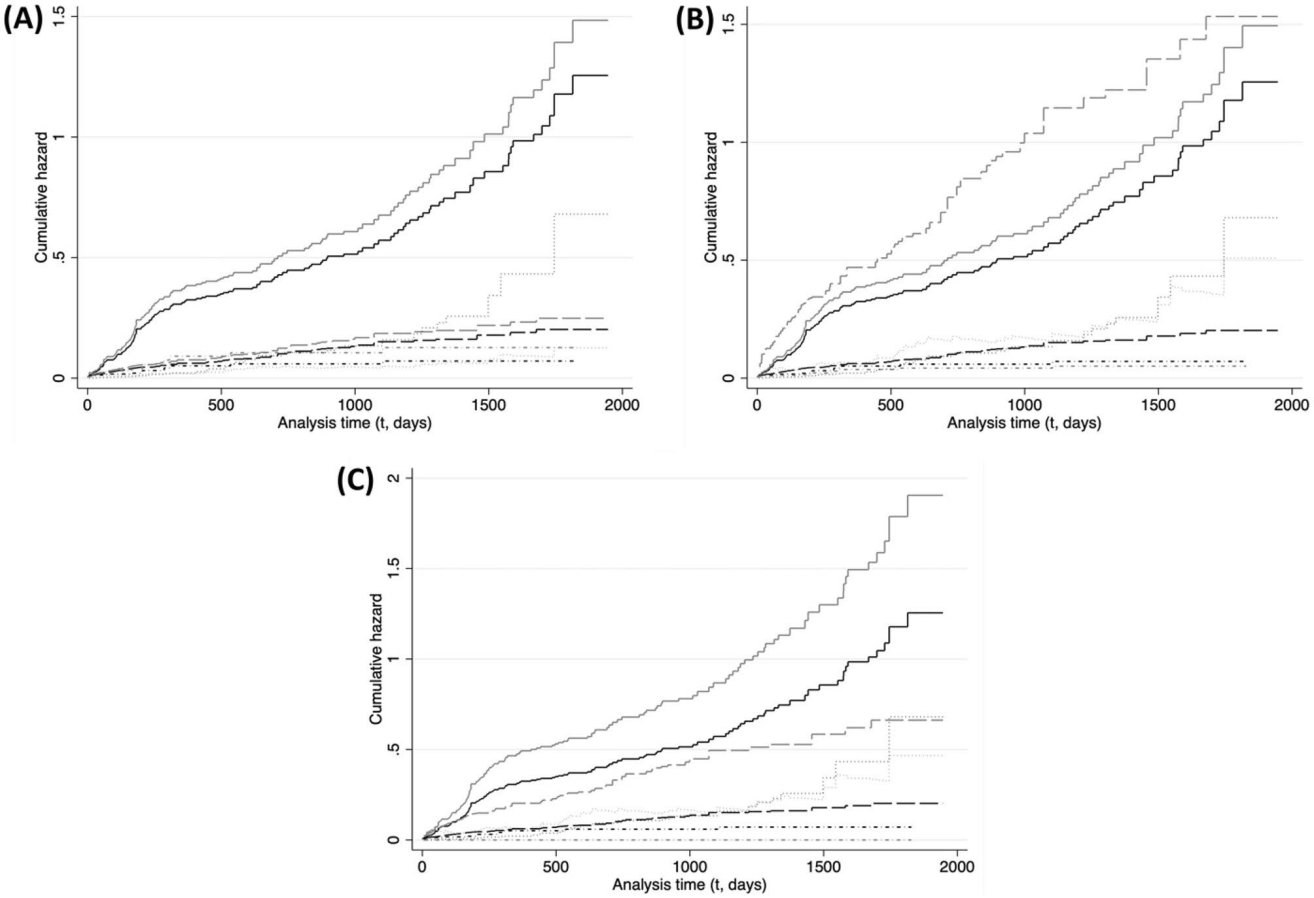
Endpoint-specific cumulative hazard over monitoring time (in days) during strict protection periods (*lib_kill* = 0) derived from endpoint-season specific hazards obtained from our preferred joint stratified Cox model (Model 3, Table 1) for n = 495 adult monitored wolves in Wisconsin, USA (1979–2012). Each panel corresponds to a season ((**A**) *hunt/hound*, (**B**) *hunt/hound/snow*, (**C**) *snow*) and illustrates the baseline (black curves) and seasonal (gray curves) cumulative hazards for our endpoints of interest: LTF (solid), reported poached (longdash), legal killing (dash-dot) and natural (dot).

**Figure 2:**
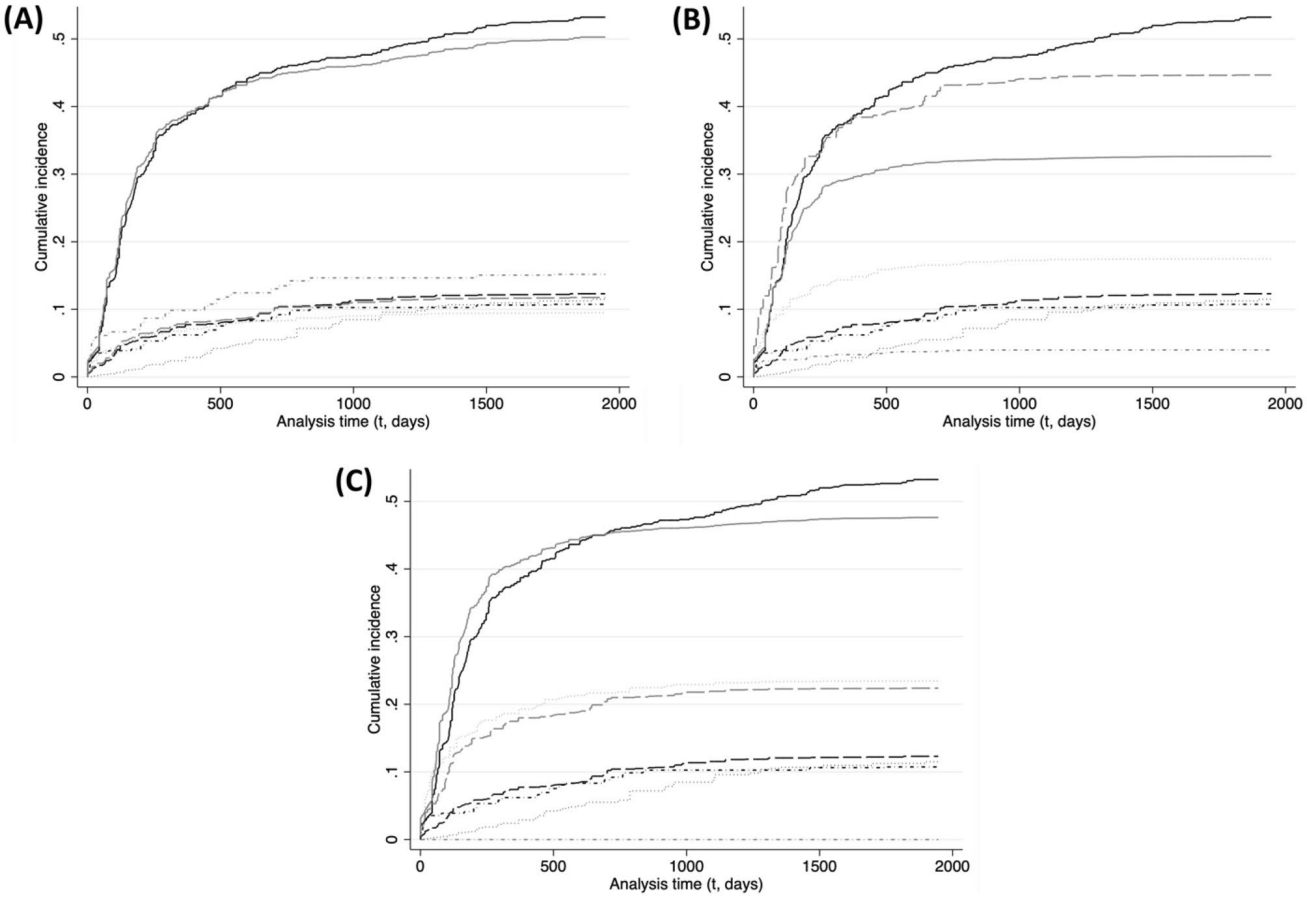
Endpoint-specific cumulative incidence curves (CIFs) over monitoring time (in days) constructing using all endpoint hazards obtained from our preferred joint stratified Cox model (Model 3, Table 1) for n = 495 adult monitored wolves in Wisconsin, USA (1979–2012). Each panel corresponds to a season ((**A**) *hunt/hound*, (**B**) *hunt/hound/snow*, (**C**) *snow*) and illustrates the baseline (black curves) and seasonal (gray curves) cumulative incidences for our endpoints of interest: LTF (solid), reported poached (longdash), legal killing (dash-dot) and natural (dot).

Below we present results of constructed cumulative hazard curves (Fig. 1, Panels A–C) and CIFs (Fig. 2, Panels A–C) using our stratified joint Cox Model 3 (Table 1). Figure 1 illustrates how endpoint-specific hazards accumulate over a wolf’s monitoring time, by season, and allow for comparing the magnitude (rather than HR) of each endpoint’s hazards. Figure 2 allows for discerning any interactions between endpoint hazards over time.

#### Baseline season (Figs. 1, 2, panels A–C)

LTF has by far the highest cumulative hazard and incidence of all endpoints throughout the season. Both hazard and incidence of other endpoints are much lower relative to LTF. The second highest cumulative hazard during the season belongs to reported poached until t = 600 (Fig. 1), when it is matched by the hazard of a natural endpoint (lower before) up to t = 1200 (Fig. 1). The hazard of a natural endpoint becomes the second highest cumulative hazard during the season at t > 1200 (Fig. 1), yet its incidence remains lower than reported poached until t = 2000, when it reaches similar levels (0.12, Fig. 2).

#### Hunt/hound season (Figs. 1, 2, panel A)

LTF remains the endpoint with the highest cumulative hazard and incidence of all endpoints (Figs. 1, 2) throughout the hunt/hound season, despite having the lowest HR increase (Table 1). The legal killing (during strict protection periods) and reported poached endpoints (both with HR > 1, Table 1) have the second largest, and similar, cumulative hazards up to t = 700 (Fig. 1), after which reported poached overtakes legal killing as the second largest cumulative hazard (despite the lower HR). However, both endpoints maintain similar levels of incidence throughout t. The increase in hazard of legal killing results in an increased incidence (0.12–0.085 = 0.35, t = 2000; Fig. 2) similar in magnitude to the observed decrease in cumulative incidence of LTF (0.562 − 0.525 = 0.037, t = 2000; Fig. 2), which suggests the decrease in LTF incidence (HR > 1, Table 1) is influenced by the increase in hazard and incidence of legal killing. This increase in legal killing hazard may also preclude higher increases of incidence of the reported poached endpoint, despite the latter also having an HR > 1 (Table 1). The cumulative hazard of a natural endpoint becomes lower than during the baseline season by t < 450 (Fig. 1), and is the lowest cumulative hazard in the season throughout t. The incidence of a natural endpoint equals that of legal killing and reported poached until t = 700 (Fig. 2), after which it becomes the lowest.

#### Hunt/hound/snow season (Figs. 1, 2, panel B)

Reported poached is the endpoint with the highest cumulative hazard throughout t, followed by LTF (Fig. 1). Indeed, the reported poached cumulative hazard is more than 1.5 times the cumulative hazard of LTF by t = 750 (0.81/0.53 = 1.52) and until t > 1200 (Fig. 1). Reported poaching also has the highest incidence throughout t. Figure 2 shows how the magnitude of the reported poached hazard results in a substantial increase in incidence of the endpoint, but also suggests the reported poaching hazard may play a role in the observed decrease in incidence of LTF (despite an HR > 1, Table 1). The third highest cumulative hazard and incidence throughout the season belongs to the natural endpoint, which is only on average about a third of the LTF cumulative hazard (Fig. 1) and half its incidence (Fig. 2). Legal killing has the lowest cumulative hazard throughout t.

#### Snow season (Figs. 1, 2, panel C)

LTF has the highest cumulative hazard and incidence throughout t. The second highest cumulative hazard belong to the reported poached endpoint, which amounts on average to half of the LTF cumulative hazard throughout t. The natural endpoint has the third highest cumulative hazard in the season but the second highest incidence, which is marginally higher than reported poached throughout t (Fig. 2). This increase in natural incidence relative to baseline, despite similar cumulative hazards, may be due in part to decreases in hazard of the unknown and legal endpoints (Table 1).

## Discussion

Time-to-event models for wild animals generally model exposure of individuals to natural conditions that may affect the risk of mortality and disappearance. Most models neglect to consider seasons of high human activity that may affect such risks, or interactions between endpoint hazards (reflected in incidences) that may illuminate ecology. For many large carnivores, which suffer from low natural mortality yet are also subject to high risk of anthropogenic mortality and poaching, seasons of anthropogenic activity may be as important as natural ones in mediating cause-specific mortality and disappearance.

Importantly, such anthropogenic seasons of higher mortality need not be specific to the animals being studied, especially if the species is controversial and much mortality illegal: our anthropogenic seasons consist of state hunting and hounding seasons for species other than wolves (i.e., deer or bear hunting, and hounding; not wolf hunting), but that mediate human activity on the landscape during those seasons. Our results support the hypothesis that increases in poaching risk during hunting seasons may be attributable to the surge of individuals with inclination to poach on the landscape^14,18,29^. Alternatively, it could also suggest enhanced criminal activity of a few poachers during the same periods. We temper this increase in poaching risk by establishing snow cover as a major environmental factor strongly associated with poaching. Moreover, our time-to-event analyses illuminate how to evaluate the effects that such anthropogenic seasons may have on risk of mortality and disappearance of monitored animals throughout their lifetime, and how considering such seasons may elucidate the mechanisms behind anthropogenic mortality and disappearance.

Additionally, our analysis period precedes and completely excludes any established public wolf hunting seasons. Hence, our modeled anthropogenic seasons represent the periods of most relevant anthropogenic activity for wolves, as hypothesized by other studies^14,29,33^ and suggested by social science studies on inclinations to poach self-reported by both deer hunters and bear hunters, as well as acceptance of poaching by hunters and farmers^30–32^.

Our analyses show increases in the hazard of disappearances of collared wolves (LTF) relative to the baseline period (which excludes environmental and anthropogenic risks) for all seasons. The highest hazard of LTF occurs during the snow season, whereas increases in hazard are lower (and similar) for the two seasons that included hounding and hunting. LTF may experience changes in hazard due to changes in the hazard of any/all of its components: migration, collar failure, or cryptic poaching.

Constant and steep increases in LTF hazard throughout a wolf’s lifetime suggests mechanisms other than migration regulating LTF hazard, given migration for adults is most frequent by yearlings and younger adults, around 1.5 to 2.2 years^34–36^. Moreover, only migration out of state would end monitoring, not routine extraterritorial movements of radio-collared wolves. That our seasonal LTF curves depict the cumulative hazards more than doubling beyond those t generally associated with dispersal (~ t < 500, given wolves were collared as adults), and that such hazards remain high throughout a wolf’s lifetime relative to other endpoints, suggests mechanisms behind LTF hazard that are additional to migration out of state. If migration had been the driving mechanism behind LTF hazard, we would also expect higher increases in hazard (more similar to the snow season) during other periods also associated with increased dispersal for adults, such as Oct–Nov^36^ within the hunt/hound and hunt/hound/snow seasons. Instead, during the latter seasons we observe smaller increases in LTF hazards, again suggesting mechanisms other than long-range movements out of state raising LTF hazard.

Although our study is unable to evaluate the contribution of collar failure to LTF hazard, we note that average and max time to LTF (t = 497, 2330 respectively) was similar to that of other anthropogenic endpoints (legal, t = 472, 2357; poached, t = 477, 2303) and much shorter than for other endpoints (collision, t = 590, 2235; natural, t = 655, 3051; unknown, t = 773, 2999) or censored observations (t = 882, 2833), which implicates causes other than battery or collar failure^14^.

As for the cryptic poaching component of the LTF hazard, the mechanism is consistent with the observed steep increase in hazard of LTF throughout a wolf’s lifetime and seasons (contrary to the natural hazard), and with the similarities in time to endpoint between LTF and other anthropogenic, intentional killing (i.e., legal killing and reported poached).

The lower increases in LTF hazard during the hunt/hound and hunt/hound/snow seasons relative to the snow season show different patterns to that of the reported poached endpoint. We hypothesize the much higher relative cumulative hazard of the LTF endpoint for all seasons except hunt/hound/snow (for which reported poached is highest) may suggests a rate of cryptic poaching that increases not only due to more cryptic poaching activity than baseline during periods of more anthropogenic activity (hunt/hound and hunt/hound/snow seasons), but also due to decreased detection of poaching on the landscape given environmental conditions during the snow season^33^. This reduced detection of cryptic poaching which increases LTF hazard during the snow season does not translate to the hunt/hound/snow season (despite similar environmental conditions) due to a surge of individuals on the landscape that result in not only more, but detectable poaching, therefore increasing the reported poached rather than the LTF hazard. This seems to resemble the pattern reported in Santiago-Ávila et al.^18^ of an increase in the hazard of reported poached relative to that of LTF during a census period in which dozens of civilian wolf-trackers went out in snow months to count wolves. Therefore, search effort and visibility due to landscape conditions are important variables to consider when designing anti-poaching interventions.

The hazard of reported poached more than doubles during the snow season relative to the baseline season, and doubles again during the hunt/hound/snow season, during which wolves are simultaneously exposed to environmental and anthropogenic conditions. The reported poached cumulative hazard during the hunt/hound/snow season is the highest of any across endpoint-seasons. These results implicate snow cover as a major factor mediating poaching activity (much lower hazard during snowless seasons), potentially by increasing wolf track detection. To those conditions, the hunt/hound/snow season may add more potential poachers or their increased killing, particularly during the (firearm) deer season, which more than doubles the snow season reported poached hazard. An important observation is that despite a decrease in incidence of LTF that season, in fact the LTF hazard increases, which points to this seasonal decrease in LTF incidence being an effect of the substantial increase in reported poaching hazard; i.e., the much higher rate of reported poached decreases LTF incidence despite an increased hazard of LTF. Therefore, we conclude that the reporting and documentation of poaching is improved when there are more people on the landscape, and worsened when there are fewer and snow cover is high.

For all anthropogenic and environmental seasons modelled, the natural endpoint shows an initial higher hazard but with a decrease in its seasonal hazard over time relative to baseline (i.e., non-proportional effects). The natural hazard is in general lowest during the hunt/hound season. For the hunt/hound/snow and snow seasons, the natural hazard is substantially lower than the LTF or reported poached endpoints. Moreover, the deceleration in the increase in natural hazards relative to the baseline period is suggestive of wolves learning to mitigate some seasonal natural hazards over their lifetime (e.g., intraspecific strife, starvation). We do not observe such a pattern with the LTF or reported poached endpoints, for which increases in hazard continue unabated over time. The difference in patterns between natural and anthropogenic endpoints suggests wolves may have difficulty and limited success in mitigating the hazard of anthropogenic killing, which is also by far the highest hazard overall. We also note that the natural hazard is lower than that for reported poached during the snow season, despite the marginally higher natural incidence, suggesting the latter could be an effect of the interaction of the natural hazard with lowered hazards from other, less prevalent endpoints (e.g., unknown, legal). The higher hazard of poaching (cryptic, through LTF, and reported) relative to other endpoints makes any possible interactions (compensatory or depensatory) among the other hazards (e.g., between natural death and legal killing) seem marginal and possibly influenced by (correlated to) fluctuations in the hazard of poaching. Hence, we caution researchers looking for compensatory or depensatory mechanisms to account for the role of poaching, including its cryptic component, first and foremost.

Our results also indicate different seasonal patterns of hazard for our natural and unknown endpoints, which suggests they should be analyzed separately (contra^29^). Failure to do so would inflate estimates of anthropogenic mortality and exaggerate the sustainability of lethal management programs that base predictions on estimates of human-caused mortality (e.g.^37^). Results for endpoints of lower prevalence, such as unknown, collisions, and (to a lesser extent) legal killing when implemented as in Wisconsin (by government agents removing suspected predators of livestock primarily), should be considered preliminary given their respective lower numbers of events per modeled covariate than those recommended to ensure accurate estimation^38,39^.

The increase in hazard of reported poached and LTF during the hunt/hound/snow season makes this season the deadliest for wolves throughout most of their adult lives (see Supplementary Material Fig. 3). The high hazards of LTF and reported poached, which are higher than all other endpoints for most seasons (hunt/hound, hunt/hound/snow and snow) and throughout t, also confirm poaching as by far the highest mortality hazard for collared adult wolves in Wisconsin throughout their lifetimes^14,18^.

Furthermore, given attitudes toward wolves became more negative among relevant demographics after wolf hunts were implemented in Wisconsin in 2012^32^, the general hazard of poaching (cryptic and reported, for all seasons) may have increased relative to our study period (when wolf hunts were not legal) despite possibly resulting in a relatively lower incidence due to the magnitude of the increase in legal killing (e.g., Wisconsin February 2021 wolf hunt^40^). Moreover, the ‘facilitated poaching’ hypothesis suggests further increases in poaching after permitting wolf hunting, trapping, and hounding (2012–2014, 2021–) relative to only permitting selected legal killing (our study period)^17,18,25^. Such an effect of public wolf-hunts would hypothetically be mediated by a policy signal that further devalues wolves or suggests overabundance.

We are not aware of effective efforts by the WDNR to mitigate poaching hazard, neither through increased enforcement nor through public education initiatives. Rather, WDNR efforts have been focused on ‘tolerance hunting’ through reducing protections, despite multiple lines of evidence pointing to such actions not decreasing and potentially increasing total (cryptic and reported) poaching hazard^14,18,25,31,32^. In other jurisdictions, such ‘tolerance killing’ is viewed skeptically as a management tool both scientifically and legally^13,41–43^. Our results underscore the need for increased protections and anti-poaching interventions to improve the wellbeing of wolves and their populations, and to reduce illegal exploitation of the public trust.

## Methods

### Data sources and preparation

We analyzed data acquired from the Wisconsin Department of Natural Resources (WDNR) which includes all collared, adult wolves monitored via telemetry (consisting almost entirely of VHF transmitters) in Wisconsin, USA between 1979 and April 14, 2012, published previously in Treves et al.^14^ and Santiago-Ávila et al.^18^ (n = 495). The dataset includes 487 collared wolves captured and monitored by the WDNR and agents, in addition to 8 wolves initially captured in MI with full monitoring history.

For those wolves monitored until death (n = 242, 49% of monitored individuals), the recorded endpoint classifies their cause of death by one of 5 mutually exclusive causes (following^14,18^): collision (with vehicles; n = 24, 5%), legal (lethal control by agency personnel; n = 32, 6%), reported poached (illegal killings reported to and evidenced by the agency; n = 88, 18%), natural (unrelated to humans, such as disease or intraspecific strife; n = 77, 16%) or unknown (uncertain cause of death; n = 21, 4%). Dead wolves were recovered via the mortality signal emitted from collars; legal killing by agents; or after reports by private citizens. We defined the date of endpoint for wolves monitored until death as their agency-recorded date of death.

In addition to wolves monitored until death, the data includes 213 wolves (43% of monitored individuals) with a ‘lost-to-follow-up’ (LTF) endpoint. LTF may occur because: (a) collars stop transmitting (i.e., mechanical failure); (b) permanent migration out of monitoring range; or, (c) cryptic poaching (i.e., concealed and undetected poaching)^17,18^. The WDNR assigned an LTF endpoint to a wolf if agency personnel was unable to detect the collar signal after various months of aerial or ground telemetry (although effort was not quantified)^14,18^. We defined the date of endpoint for LTF wolves as the last date of telemetry contact with them. There were 33 LTF wolves (15% of LTF and 7% of collared) that were later recovered, a third of them poached (n = 11). Our main results classify these as LTF, but we include results for a separate endpoint classification of these 33 wolves as ‘known-LTF’ in supplementary materials. We censored those individuals that survived until the end of the monitoring period (April 15, 2012, n = 40). Our LTF endpoint is conservative given we censored, rather than impute (as in Santiago-Ávila et al. 2020), the fates of n = 26 wolves that disappeared sometime between December 31st, 2011 and April 14th, 2012 and lacked subsequent monitoring or endpoint data in reports between 2012 and 2013 (see Supp Data S2 in Ref.^18^). Simulations suggest at least some of these latter wolves may have gone LTF in the winter of 2011–2012^18^.

We include two external time-dependent covariates in our statistical models (see below), which are variables that change value at specific dates due to external events, such as a change in season or policy. To include those variables, we split each wolf’s monitoring history into time intervals at each specific date of change of that variable so that its value remains constant for each interval. Therefore, each time interval reflects the type of period each wolf was exposed to, and the specific dates during which s/he was exposed.

Our main covariate of interest, risk_season, is a four-level categorical variable defining intra-wolf-year periods (wolf-year = April 15th to April 14th) characterized by specific anthropogenic (i.e., hounding and hunting seasons for deer and black bear) and environmental (i.e., snow cover) factors, their overlap, and absence (Table 2). We used specific dates to split each wolf-year in our study period (1979–2012) into four distinct seasons. Our baseline period (risk_season = 0) refers to April 15th to June 30th (or to July 9th from 1991 to 2012) and is characterized by the absence of the anthropogenic and environmental conditions present in the other variable levels (i.e., no hounding, no white-tailed deer or black bear hunting, no snow cover). Our hounding and hunting season without snow cover (risk_season = 1, ‘hunt/hound’), runs from July 1st (July 10th from 1991 to 2012) to Nov 14th. In WI, use of hounds for bear hunting was legalized in 1963 and bear dog training was allowed starting July (1st or 10th) until August 31st. Deer and bear seasons start soon thereafter, in early to mid-September, with the deer season running through the first Sunday in January for most counties (in some counties, the deer season extends to January 31st). Our hounding and hunting season with snow cover (risk_season = 2, ‘hunt/hound/snow’) starts Nov 15th and runs through the first Sunday in January, when deer hunting season ends for most counties in WI. Average annual duration of snow cover extends to > 140 days along Lake Superior (http://aos.wisc.edu/), and most occupied wolf range is in northern Wisconsin. To this data, we added statewide monthly average snowfall (1975–2011) from the WI State Climatology Office, modeling snow cover to include months with an average snow cover of > 1 inch (November through May). Considering both data sources, starting the period on November 15th (average 5.31 in; October, 0.63 in) allowed us to model 151 days of snow cover up to April 14th (average 2.88 in; May, 0.19 in), the end of the wolf-year. Lastly, our snow cover season without hounding or hunting (risk_season = 3, ‘snow’) runs from the Monday after the first Sunday in January (when deer season closes for most WI counties), until April 14th, as per our snow cover modeling. A breakdown of events per endpoint and time at risk by season is provided in Supplementary Material Table 2.

**Table 2:** Intra-wolf-year (April 15th–April 14th) seasons (*risk_season*) characterized by the absence (*baseline* level), presence or overlap of anthropogenic and environmental factors mediating endpoint-specific risk.

Season starts	Season ends	Season (risk_season)
April 15th	June 31st (pre–1991) or July 9th (1991 onward)	‘Baseline’; no hounding/hunting/snow (0)
July 1st (pre–1991) OR July 10th (1991 onward)	November 14th	‘Hunt/hound’ (1)
November 15th	1st Sunday in January	‘Hunt/hound/snow’ (2)
Monday after 1st Sunday in January	April 14th	‘Snow’ (3)

We also model policy protection periods following Santiago-Ávila et al.^18^ (lib_kill, where 1 = reduced protections, i.e., liberalized killing; and 0 = full protection), and include it as a stratifying variable in our statistical models, given evidence of endpoint-specific and sometimes non-proportional effects. In WI, gray wolves were exposed exclusively to full protections under the Endangered Species Act (ESA) from 1979 to March 31, 2003. From April 2003 to 2012, wolves in WI (and MI) were exposed to 11 alternating, sequential and mutually exclusive periods of reduced and restored protections that liberalized and restricted wolf-killing, respectively (Ref.^44^, Supplementary Material Table 2). Periods of reduced protections and liberalized killing (including periods during which permits for ‘take’ were issued, as well as periods of ‘down-’ and ‘de-listing’ from the ESA) were characterized by an announcement of policy change reducing constraints for managers or landowners to kill wolves in response to perceived or actual conflicts, most notably wolf predation on domestic animals.

### Statistical tests

Our methods exploit the survival history of collared, monitored adult wolves, measured in days (t), from date of capture and collaring to date of endpoint (i.e., death by multiple causes (see “Data sources and preparation” section) or disappearance). Survival analysis estimates ‘time-to-event’ functions; i.e., the probability of observing a time interval (T), from beginning of monitoring to endpoint, greater than some stated value ‘t’, $\mathrm{S}\left(\mathrm{t}\right)=\mathrm{P}(\mathrm{T}>\mathrm{t})$. Such techniques also allow for estimating (endpoint-specific) hazard functions, ${\mathrm{h}}_{\mathrm{k}}\left(\mathrm{t}\right)$; the instantaneous rate of occurrence of an endpoint (k) conditional on not experiencing any endpoint until that time^45–47^. Semi-parametric, Cox proportional hazard models allow for the estimation of how endpoint-specific hazards change as a function of survival (i.e., monitoring) time and a set of covariates $\mathrm{S}\left(\mathrm{t}\right)={\mathrm{e}}^{-{\mathrm{h}}_{\mathrm{k}}(\mathrm{t},\mathrm{x},\upbeta )}$, where x refers to a vector of covariates and β to its parameter estimates. Cox models estimate these covariate effects on endpoint-specific hazard(s) as ${\mathrm{h}}_{\mathrm{k}}\left(\mathrm{t}\right)= {\mathrm{h}}_{0\mathrm{k}}(\mathrm{t}){\mathrm{e}}^{({\upbeta }_{1}{\mathrm{x}}_{1}+\dots +{\upbeta }_{\mathrm{j}}{\mathrm{x}}_{\mathrm{j}})}$, where ${\mathrm{h}}_{0\mathrm{k}}(\mathrm{t})$ is an unestimated baseline hazard function (i.e., semi-parametric), and β_j_ represent the estimates of HRs for each covariate x_j_ (HR > 1 is interpreted as an increase, and HR < 1 as a decrease, in hazard).

We employed the Lunn and McNeil^48^ data augmentation technique (by k endpoints) to build stratified (by endpoint) joint Cox proportional hazard models to simultaneously estimate endpoint-specific changes in HRs for each endpoint-season interaction. In using a Cox model, we assume that the endpoint and time-to-endpoint for each wolf is independent of other wolves’ (i.e., one wolf’s monitoring history and endpoint does not inform others). Because we split the monitoring history of wolves into ‘spells’ for inclusion of time-dependent covariates (see “Data sources and preparation” section), we cluster analyses by following^49^. We also assume censoring is independent of other endpoints, as we explicitly account for LTF as a separate endpoint given evidence it contains an unaccounted-for source of mortality^14,17,18,29^. We evaluate compliance with our proportionality assumptions using Schoenfeld residuals^46,47,50^. We control for non-proportionality of endpoint-season interactions, when necessary, through the inclusion of time-varying coefficients (tvc) for the respective interaction(s). A tvc is an interaction of a parameter with a function of analysis time (t), in our case, $\mathrm{ln}(\mathrm{t})$, to model the change in the main endpoint-season parameter’s effect over time. We selected the preferred Cox model considering Akaike’s Information Criterion (AIC) and weights, Bayesian Information Criterion (BIC), and compliance with Cox model assumptions.

We then proceed with a competing risk approach by using endpoint-season specific parameter estimates from the best stratified joint Cox model to construct cumulative incidence curves (CIFs) for each endpoint and season. Competing risk approaches focus on the estimation of endpoint-specific CIFs, defined by the failure probability $\mathrm{Prob}(\mathrm{T}\le \mathrm{t},\mathrm{D}=\mathrm{k})$; i.e., the cumulative probability of an endpoint, k, occurring over time in the presence of all other competing endpoints^45,51,52^. These analyses account for the CIF of any endpoint being a function of all endpoint-specific hazards, ${\mathrm{h}}_{\mathrm{k}}\left(\mathrm{t}\right)$, thus accounting for the rate of occurrence of that endpoint in addition to how other endpoints influence it^53^. Thus, joint analysis of hazards and incidence is essential for discerning interactions between endpoint hazards and how they are reflected on each endpoint’s incidence.

Consistent with rigorous approaches to competing risk analyses, we present and discuss results for our best performing stratified joint Cox model, by endpoint and season, as well as endpoint-specific CIFs, by season, and synthesize findings^39,45,51,53^. We conducted all statistical analyses in Stata 16 (StataCorp LLC, College Station, TX, 2019).

## Supplementary Information

Supplementary Information 1.

Supplementary Information 2.

Supplementary Information 3.
