# Supplementary material for: Poaching of protected wolves fluctuated seasonally and with non-wolf hunting
Source: Sci Rep. 2022 Feb 2;12:1738. doi: 10.1038/s41598-022-05679-w (PMC8810790; doi:10.1038/s41598-022-05679-w)
Supplement: Supplementary file 3 — Supplementary Information 3. [file 41598_2022_5679_MOESM3_ESM.docx]

**Poaching of protected wolves fluctuated seasonally and with non-wolf hunting**

Francisco J. Santiago-Ávila^*^ & Adrian Treves

Nelson Institute for Environmental Studies, University of Wisconsin – Madison

*corresponding author: [santiagoavil@wisc.edu](mailto:santiagoavil@wisc.edu)

Supplementary Tables 1-7:

| **Endpoint** | ***Baseline*** | ***Hunt/hound*** | ***Hunt/hound/***  ***snow*** | ***Snow*** | ***Total*** |
| --- | --- | --- | --- | --- | --- |
| *LTF* | 27 | 89 | 34 | 63 | 213 |
| *legal* | 9 | 21 | 2 | 0 | 32 |
| *nonhuman* | 6 | 20 | 19 | 32 | 77 |
| *reported poached* | 8 | 20 | 36 | 24 | 88 |
| *unknown* | 4 | 12 | 2 | 3 | 21 |
| *collision* | 2 | 14 | 1 | 7 | 24 |
| **Total** | **56** | **176** | **94** | **129** | **455** |
| **Time at risk (t, days)** | **52,213** | **116,791** | **40,225** | **69,805** | **279,034** |

**Table 1.** Number of events (unique wolf IDs) per endpoint and season (*risk_season* variable, see Methods). Wolves that survived to the end of the study period are omitted here and censored at the end of the study period (n=40).

| **Period start (dd/mm/yyyy)** | **Period end (dd/mm/yyyy)** | **Federal status** | **Policy period****  **(*lib_kill*)** |
| --- | --- | --- | --- |
| 15/04/1994 | 31/03/2003 | Listed as endangered | full protections (0) |
| 01/04/2003 | 30/01/2005 | Down-listed to threatened | liberalized killing (1) |
| 31/01/2005 | 31/03/2005 | Relisted | full protections (0) |
| 01/04/2005 | 13/09/2005 | Sub-permit for killing issued | liberalized killing (1) |
| 14/09/2005 | 23/04/2006 | Sub-permit rescinded | full protections (0) |
| 24/04/2006* | 31/07/2006 | Sub-permit for killing issued | liberalized killing (1) |
| 01/08/2006 | 11/03/2007 | Sub-permit rescinded | full protections (0) |
| 12/03/2007 | 28/09/2008 | Delisted | liberalized killing (1) |
| 29/09/2008 | 03/05/2009 | Relisted | full protections (0) |
| 04/05/2009 | 30/06/2009 | Delisted | liberalized killing (1) |
| 01/07/2009 | 26/01/2012 | Relisted | full protections (0) |
| 27/01/2012 | 14/04/2012 | Delisted | liberalized killing (1) |

**Table 2.** Periods of wolf policy changes in Wisconsin and Michigan during our study period (*lib_kill* variable), by policy period in Wisconsin, ﻿derived from Refsnider (2009), ESA sec. 4 10(a)(1)(A) and Humane Society of the U.S. et al. v. Jewell (U.S. District Court, D.C., 5 1:13-cv-00186-BAH Document 52, 2014). *WI and MI are identical except for the sub-permit issuance on 6 May 2006 to Michigan instead of issuance on 24 April 2006 to Wisconsin*.* **Killing a wolf that posed a threat to human safety was always allowed under ESA sec.11(a)(3).

| **Model selection criteria** | **Model 1** | **Model 2** | **Model 3 (BEST)** |
| --- | --- | --- | --- |
| *Log likelihood* | -2114.944 | -2106.737 | -2107.673 |
| *AIC* | 4263.888 | 4257.474 | 4257.347 |
| *relative likelihood* | 6.541 | 0.127 | 0.000 |
| *AIC weights* | 0.038 | 0.938 | 1.000 |
| *BIC* | 4401.188 | 4435.157 | 4426.954 |
| *Model description* | no tvcs | Model 1 + potential tvcs | Model 2 excluding *hunt/hound*ltf* tvc |

**Table 3.** Model selection statistics for stratified joint Cox models for analyses of hazards using Lunn & McNeil’s data augmentation Method B. We present log-likelihood(ll), AIC and BIC statistics for all models (1-3), along with a brief model description. Model statistics along with significance of tvc parameters (see Table 2 and Tables 4-6) suggest Model 3 was the best and most parsimonious model.

| **SEASON** | ***HUNT/HOUND*** | | | | ***HUNT/HOUND/SNOW*** | | | | ***SNOW*** | | | |
| --- | --- | --- | --- | --- | --- | --- | --- | --- | --- | --- | --- | --- |
| *ENDPOINT* | **HR (se)** | | **95 CI** | | **HR (se)** | | **95 CI** | | **HR (se)** | | **95 CI** | |
| *LTF* | 1.18 | 0.72 | | 1.95 | 1.19 | 0.65 | | 2.19 | 1.52 | 0.90 | | 2.55 |
|  | (-0.3) |  | |  | (-0.37) |  | |  | (-0.4) |  | |  |
| *legal* | 1.78 | 0.79 | | 4.05 | 0.72 | 0.11 | | 4.71 | 0.00 | 0.00 | | 0.00 |
|  | (-0.75) |  | |  | (-0.69) |  | |  | (.) |  | |  |
| *reported poached* | 1.23 | 0.52 | | 2.91 | 7.58*** | 3.19 | | 17.99 | 3.27*** | 1.36 | | 7.86 |
|  | (-0.54) |  | |  | (-3.34) |  | |  | (-1.46) |  | |  |
| *nonhuman* | 1.34 | 0.50 | | 3.62 | 3.69** | 1.21 | | 11.25 | 3.65** | 1.09 | | 12.23 |
|  | (-0.68) |  | |  | (-2.1) |  | |  | (-2.25) |  | |  |
| *unknown* | 1.01 | 0.29 | | 3.56 | 0.77 | 0.07 | | 8.46 | 1.01 | 0.25 | | 4.03 |
|  | (-0.65) |  | |  | (-0.94) |  | |  | (-0.71) |  | |  |
| *collision* | 2.61 | 0.49 | | 14.06 | 0.23 | 0.02 | | 3.00 | 1.17 | 0.19 | | 7.27 |
|  | (-2.24) |  | |  | (-0.3) |  | |  | (-1.09) |  | |  |

**Table 4.** Hazard ratio (HR) point estimates from the stratified (by endpoint and protection period) joint Cox Model 1 (our initial model, without tvcs) for n=495 monitored adult wolves, by endpoint and season. We present HRs and compatibility intervals (95 CI) for all endpoint-season interactions relative to a baseline season. Note: * p<0.10, ** p<.05, *** p<0.01.

| **Parameter (endpoint per season)** | **rho** | **chi2** | **df** | **Prob>c^2^** |
| --- | --- | --- | --- | --- |
| ***HUNT/HOUND*** |  |  |  |  |
| *LTF* | -0.07194 | 2.38 | 1 | 0.123 |
| *legal* | 0.05599 | 1.31 | 1 | 0.2522 |
| *reported poached* | 0.04518 | 0.82 | 1 | 0.3657 |
| *nonhuman* | -0.13716 | 9.94 | 1 | 0.0016* |
| *unknown* | 0.0249 | 0.44 | 1 | 0.5077 |
| *collision* | -0.03624 | 0.65 | 1 | 0.4198 |
| ***HUNT/HOUND/SNOW*** |  |  |  |  |
| *LTF* | -0.05669 | 1.61 | 1 | 0.2051 |
| *legal* | -0.04358 | 0.89 | 1 | 0.3443 |
| *reported poached* | -0.0307 | 0.47 | 1 | 0.491 |
| *nonhuman* | -0.1185 | 10.32 | 1 | 0.0013* |
| *unknown* | -0.07254 | 6.58 | 1 | 0.0103* |
| *collision* | 0.03395 | 0.58 | 1 | 0.4478 |
| ***SNOW*** |  |  |  |  |
| *LTF* | -0.0423 | 0.71 | 1 | 0.4007 |
| *legal* | . | . | 1 | . |
| *reported poached* | -0.04843 | 1.14 | 1 | 0.2862 |
| *nonhuman* | -0.09422 | 9.62 | 1 | 0.0019* |
| *unknown* | -0.02747 | 0.42 | 1 | 0.5145 |
| *collision* | 0.0141 | 0.1 | 1 | 0.7577 |
| **Global test** |  | **34.58** | **17** | **0.0071** |

**Table 5.** Test of proportional hazards assumptions (Chi-squared, χ^2^) for parameters in the stratified joint Cox Lunn & McNeil Model 1 (Table 4), used for evaluating proportionality assumptions (measured using ln(t), see Methods). The tests suggest potential non-proportionality present in the tvcs included in Model 2 (identified with a *). The tvcs in Model 3, the preferred stratified joint Cox hazards model, appropriately model the observed non-proportionality through interactions of these endpoint-covariate combinations with analysis time (i.e., tvc) (Table 2).

| **SEASON** | ***HUNT/HOUND*** | | | | ***HUNT/HOUND/SNOW*** | | | | ***SNOW*** | | | |
| --- | --- | --- | --- | --- | --- | --- | --- | --- | --- | --- | --- | --- |
| *ENDPOINT* | **HR (se)** | | **95 CI** | | **HR (se)** | | **95 CI** | | **HR (se)** | | **95 CI** | |
| *LTF* | 2.48 | 0.80 | | 7.71 | 1.15 | 0.62 | | 2.13 | 1.47 | 0.88 | | 2.46 |
|  | (-1.44) |  | |  | (-0.36) |  | |  | (-0.39) |  | |  |
| *legal* | 1.78 | 0.79 | | 4.05 | 0.72 | 0.11 | | 4.71 | 0.00 | 0.00 | | 0.00 |
|  | (-0.75) |  | |  | (-0.69) |  | |  | (.) |  | |  |
| *reported poached* | 1.23 | 0.52 | | 2.91 | 7.58*** | 3.19 | | 17.99 | 3.27*** | 1.36 | | 7.86 |
|  | (-0.54) |  | |  | (-3.34) |  | |  | (-1.46) |  | |  |
| *nonhuman* | 238.98*** | 8.61 | | 6630.66 | 392.14*** | 11.29 | | 13614.75 | 623.97*** | 7.00 | | 55655.22 |
|  | (-405.19) |  | |  | (-709.72) |  | |  | (-1429.7) |  | |  |
| *unknown* | 1.20 | 0.33 | | 4.44 | 7671.73*** | 15.11 | | 3894537.72 | 0.66 | 0.13 | | 3.32 |
|  | (-0.8) |  | |  | (-24384.75) |  | |  | (-0.54) |  | |  |
| *collision* | 2.61 | 0.49 | | 14.06 | 0.23 | 0.02 | | 3.00 | 1.17 | 0.19 | | 7.27 |
|  | (-2.24) |  | |  | (-0.3) |  | |  | (-1.09) |  | |  |
| tvc - *(ln(t))* |  |  | |  |  |  | |  |  |  | |  |
| *LTF* | 0.86 | 0.70 | | 1.06 | - | - | | - | - | - | | - |
|  | (-0.09) |  | |  | - |  | |  | - |  | |  |
| *nonhuman* | 0.39*** | 0.23 | | 0.66 | 0.44*** | 0.25 | | 0.77 | 0.41** | 0.20 | | 0.82 |
|  | (-0.11) |  | |  | (-0.13) |  | |  | (-0.14) |  | |  |
| *unknown* | - | - | | - | 0.17*** | 0.05 | | 0.55 | - | - | | - |
|  | - |  | |  | (-0.1) |  | |  | - |  | |  |

**Table 6.** Hazard ratio (HR) point estimates from the stratified (by endpoint and protection period) joint Cox Model 2 (including all potential tvc parameters) for n=495 monitored adult wolves, by endpoint and season. We present HRs and compatibility intervals (95 CI) for all endpoint-season interactions relative to a baseline season. Model 3 drops the LTF tvc given lack of evidence of non-proportionality. Note: * p<0.10, ** p<.05, *** p<0.01.

| **SEASON** | ***HUNT/HOUND*** | | | | ***HUNT/HOUND/SNOW*** | | | | ***SNOW*** | | | |
| --- | --- | --- | --- | --- | --- | --- | --- | --- | --- | --- | --- | --- |
| *ENDPOINT* | **HR (se)** | | **95 CI** | | **HR (se)** | | **95 CI** | | **HR (se)** | | **95 CI** | |
| *LTF* | 1.25 | 0.73 | | 2.16 | 1.23 | 0.63 | | 2.40 | 1.51 | 0.86 | | 2.66 |
|  | (-0.35) |  | |  | (-0.42) |  | |  | (-0.44) |  | |  |
| *legal* | 1.78 | 0.79 | | 4.05 | 0.72 | 0.11 | | 4.71 | 0.00 | 0.00 | | 0.00 |
|  | (-0.75) |  | |  | (-0.69) |  | |  | (.) |  | |  |
| *reported poached* | 1.23 | 0.52 | | 2.91 | 7.58*** | 3.19 | | 17.99 | 3.27*** | 1.36 | | 7.86 |
|  | (-0.54) |  | |  | (-3.34) |  | |  | (-1.46) |  | |  |
| *nonhuman* | 238.98*** | 8.61 | | 6630.66 | 392.14*** | 11.29 | | 13614.75 | 623.97*** | 7.00 | | 55655.22 |
|  | (-405.19) |  | |  | (-709.72) |  | |  | (-1429.7) |  | |  |
| *unknown* | 1.20 | 0.33 | | 4.44 | 7671.73*** | 15.11 | | 3894537.72 | 0.66 | 0.13 | | 3.32 |
|  | (-0.8) |  | |  | (-24384.75) |  | |  | (-0.54) |  | |  |
| *collision* | 2.61 | 0.49 | | 14.06 | 0.23 | 0.02 | | 3.00 | 1.17 | 0.19 | | 7.27 |
|  | (-2.24) |  | |  | (-0.3) |  | |  | (-1.09) |  | |  |
| *known LTF* | 9.99* | 0.87 | | 114.12 | 0.80 | 0.18 | | 3.52 | 1.25 | 0.35 | | 4.50 |
|  | (-12.41) |  | |  | (-0.61) |  | |  | (-0.82) |  | |  |
| tvc - *(ln(t))* |  |  | |  |  |  | |  |  |  | |  |
| *nonhuman* | 0.39*** | 0.23 | | 0.66 | 0.44*** | 0.25 | | 0.77 | 0.41** | 0.20 | | 0.82 |
|  | (-0.11) |  | |  | (-0.13) |  | |  | (-0.14) |  | |  |
| *unknown* | - | - | | - | 0.17*** | 0.05 | | 0.55 | - | - | | - |
|  | - |  | |  | (-0.1) |  | |  | - |  | |  |
| *known LTF* | 0.62** | 0.40 | | 0.96 | - | - | | - | - | - | | - |
|  | (-0.14) |  | |  | - |  | |  | - |  | |  |

**Table 7.** Hazard ratio (HR) point estimates from the stratified (by endpoint and protection period) joint Cox Model 3 (Table 2) with n=33 recovered LTF wolves (‘known-LTF’) reclassified as a separate endpoint (see Methods). We present HRs and compatibility intervals (95 CI) for all endpoint-season interactions relative to a baseline season. Note: * p<0.10, ** p<.05, *** p<0.01.

Supplementary Figures 1-3:


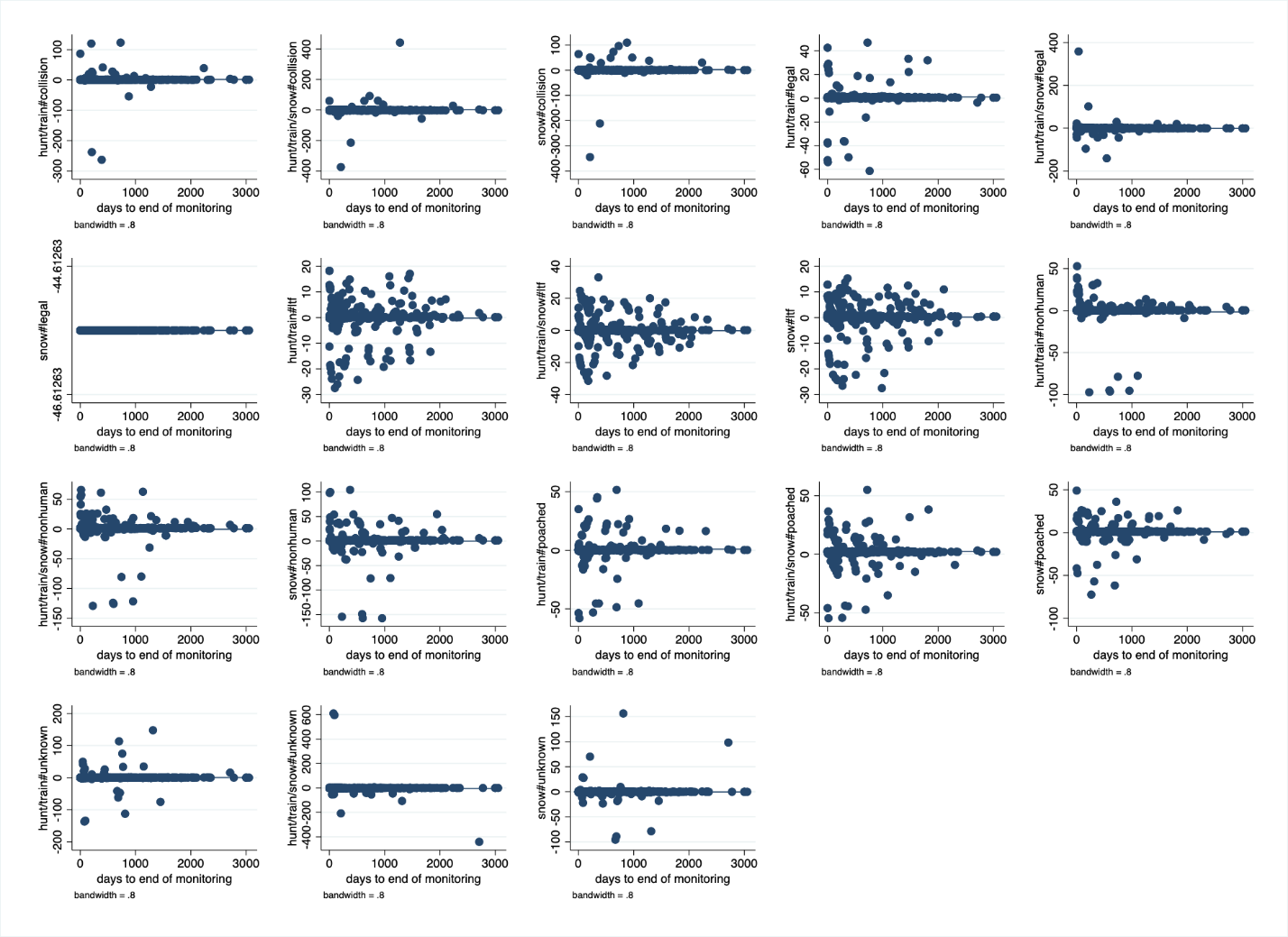


**Figure 1**. Schoenfeld residual scatterplots for each endpoint-season combination in Cox Model 1.


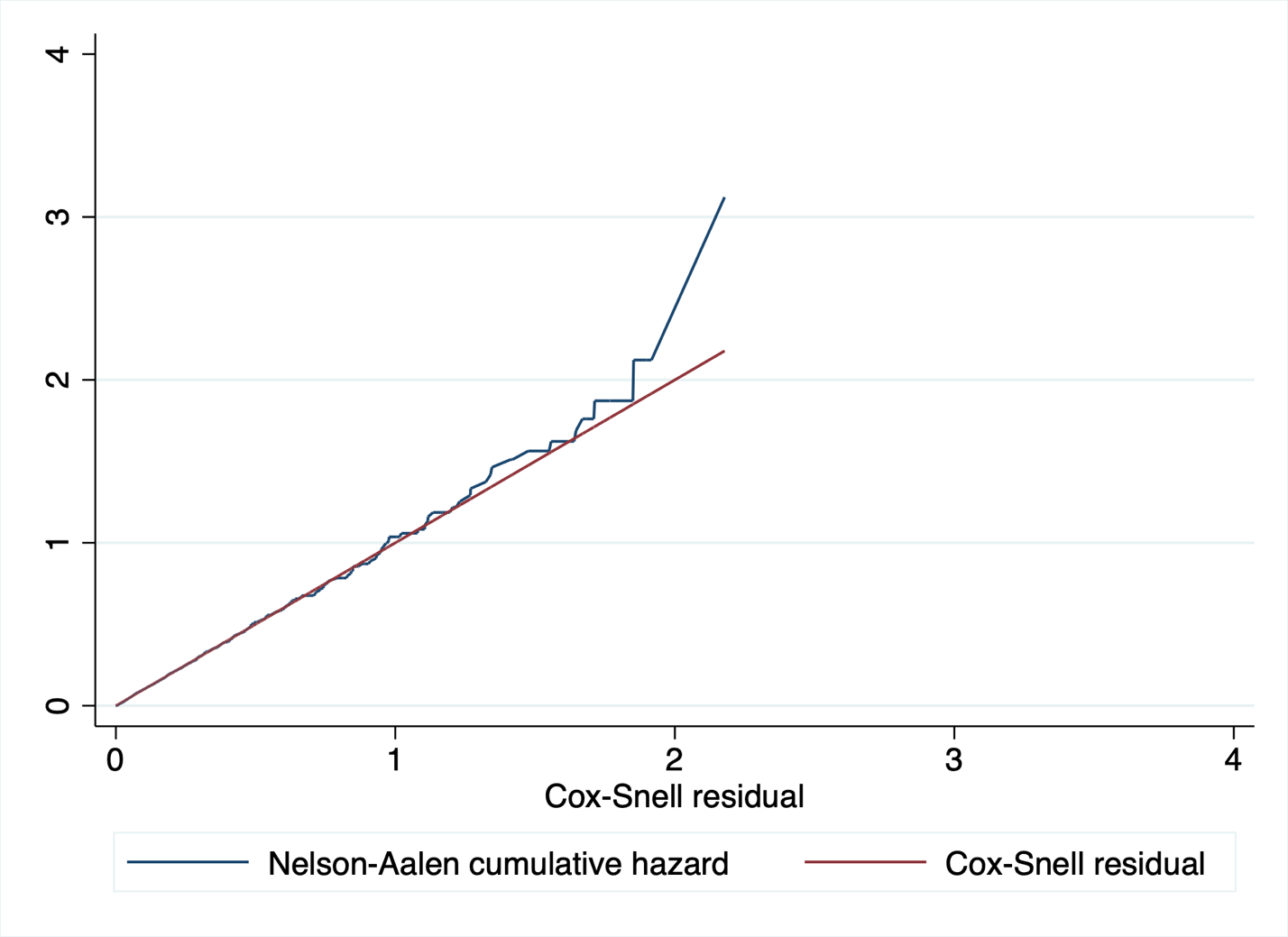


**Figure 2.** Cox-Snell generalized residuals (red lines, on x axis) used for evaluating the goodness of fit of stratified joint Cox Model 1. The Nelson-Aalen cumulative hazard (blue lines, on y axis) follows closely the 45˚ (red) line of Cox-Snell residuals and show overall consistency with the latter, although with some divergence at large values (common when censoring data).


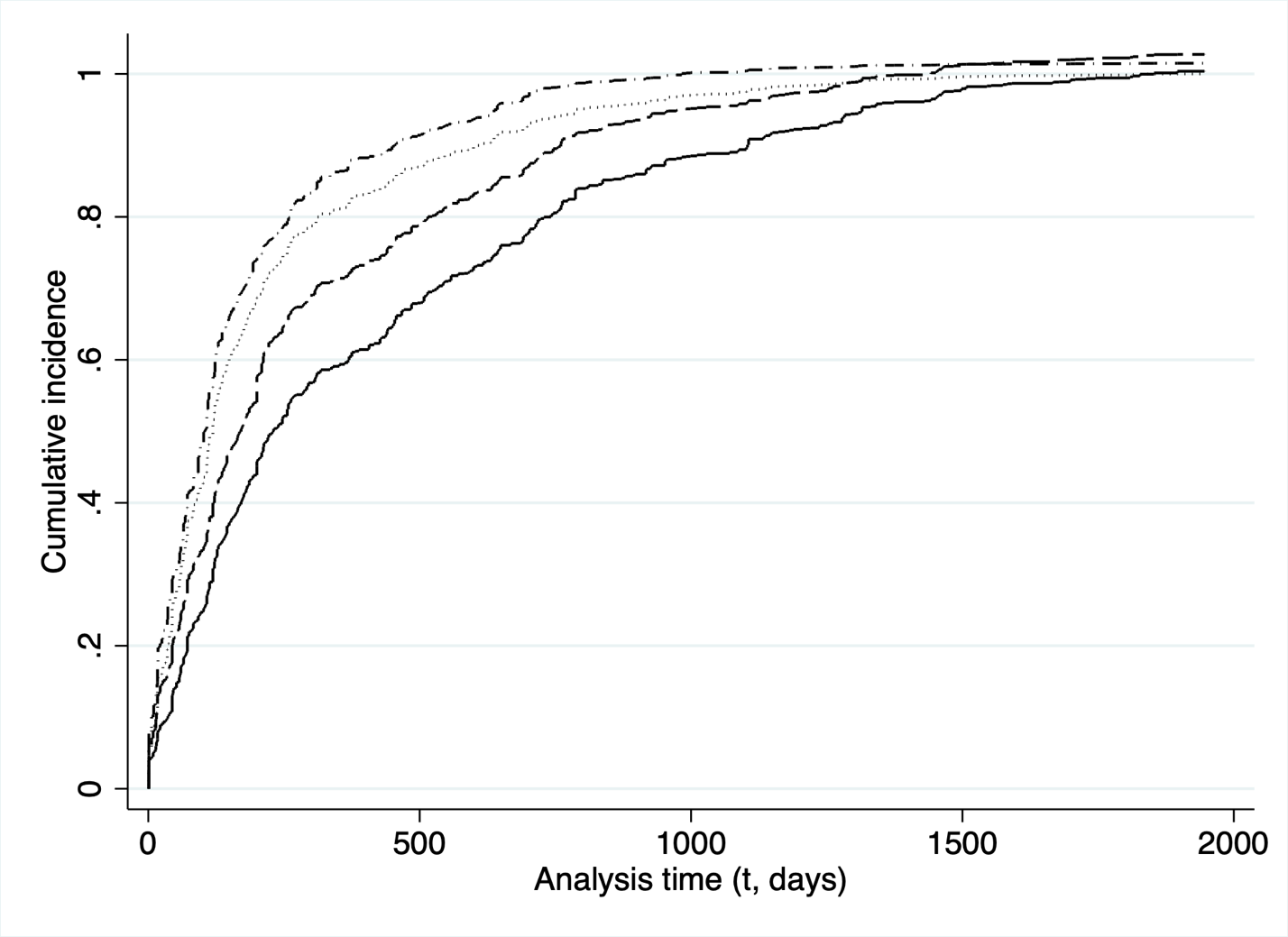


**Figure 3.** Seasonal cumulative incidence curves (CIFs) for all endpoints constructing using all endpoint hazards obtained from our preferred joint stratified Cox model (Model 3, Table 2) for n=495 adult monitored wolves in Wisconsin, USA (1979-2012): *baseline* (solid), *hunt/hound* (longdash), *hunt/hound/snow* (dash-dot) and *snow* (dot).

Statistical code for all analyses conducted in STATA

******************************************************************************

****CREATING VARIABLE FOR MUTUALLY EXCLUSIVE INTRA-YEAR PERIODS****

use "Supp_Dataset.dta", replace

*stset date_endpoint, failure(cause_endpoint2==2 3 4 5 6 7) exit(failure) origin(time capture_date) id(wolf_ID)

rename cause_endpoint2 old_endpoint

tab old_endpoint cause_endpoint

encode cause_endpoint, gen(cause_endpoint_enc)

label var cause_endpoint_enc "coded endpoint"

order cause_endpoint_enc, after(cause_endpoint)

tab cause_endpoint cause_endpoint_enc

expand 6

by wolf_ID, sort: gen cause_endpoint2= _n+1

order cause_endpoint2, after(cause_endpoint_enc)

tab cause_endpoint2 cause_endpoint_enc

/*Generating cause of endpoint binary variables*/

gen collision = cause_endpoint2==2

gen legal = cause_endpoint2==3

gen ltf = cause_endpoint2==4

gen nonhuman = cause_endpoint2==5

gen poached = cause_endpoint2==6

gen unknown = cause_endpoint2==7

gen event = (cause_endpoint2==cause_endpoint_enc)

gen censored = cause_endpoint_enc==1

*Generate ID var for each expanded record (wolf_ID - [1-6]) (CLUSTER FOR THIS INSTEAD OF wolf_ID)

gen wolf_ID_exp=wolf_ID+"-"+string(cause_endpoint2, "%02.0f")

order wolf_ID_exp, after(wolf_ID)

*Checking stset

stset date_endpoint, failure(event) exit(failure) origin(time capture_date) id(wolf_ID_exp)

*****TIME-SPLITTING for time-dependent variables and 'spells'*****

stsplit year_split, at(7129 7258 7310 7410 7495 7624 7674 7775 7860 7989 8038 8140 8225 8354 8402 8505 8590 8719 8773 8871 8956 9085 9137 9236 9321 9450 9501 9601 9686 9815 9865 9966 10051 10180 10229 10332 10417 10546 10600 10697 10782 10911 10964 11062 11147 11276 11328 11427 11503 11641 11692 11793 11869 12007 12056 12158 12234 12372 12420 12523 12599 12737 12791 12888 12964 13102 13155 13254 13330 13468 13519 13619 13695 13833 13883 13984 14060 14198 14247 14349 14425 14563 14611 14715 14791 14929 14982 15080 15156 15294 15346 15445 15521 15659 15710 15810 15886 16024 16074 16176 16252 16390 16438 16541 16617 16755 16809 16906 16982 17120 17173 17271 17347 17485 17537 17637 17713 17851 17901 18002 18078 18216 18265 18367 18443 18581 18629 18732 18808 18946 18992 19095 19098) after(capture_date==1/1/1960)

**Generating intra-year periods time-dep binary variable

gen risk_season = 0

label var risk_season "intra-year periods"

replace risk_season = 1 if year_split==7129 | year_split==7495 | year_split==7860 | year_split==8225 | year_split==8590 | year_split==8956 | year_split==9321 | year_split==9686 | year_split==10051 | year_split==10417 | year_split==10782 | year_split==11147 | year_split==11503 | year_split==11869 | year_split==12234 | year_split==12599 | year_split==12964 | year_split==13330 | year_split==13695 | year_split==14060 | year_split==14425 | year_split==14791 | year_split==15156 | year_split==15521 | year_split==15886 | year_split==16252 | year_split==16617 | year_split==16982 | year_split==17347 | year_split==17713 | year_split==18078 | year_split==18443 | year_split==18808 | year_split==19098

replace risk_season = 2 if year_split==7258 | year_split==7624 | year_split==7989 | year_split==8354 | year_split==8719 | year_split==9085 | year_split==9450 | year_split==9815 | year_split==10180 | year_split==10546 | year_split==10911 | year_split==11276 | year_split==11641 | year_split==12007 | year_split==12372 | year_split==12737 | year_split==13102 | year_split==13468 | year_split==13833 | year_split==14198 | year_split==14563 | year_split==14929 | year_split==15294 | year_split==15659 | year_split==16024 | year_split==16390 | year_split==16755 | year_split==17120 | year_split==17485 | year_split==17851 | year_split==18216 | year_split==18581 | year_split==18946

replace risk_season = 3 if year_split==6946 | year_split==7310 | year_split==7674 | year_split==8038 | year_split==8402 | year_split==8773 | year_split==9137 | year_split==9501 | year_split==9865 | year_split==10229 | year_split==10600 | year_split==10964 | year_split==11328 | year_split==11692 | year_split==12056 | year_split==12420 | year_split==12791 | year_split==13155 | year_split==13519 | year_split==13883 | year_split==14247 | year_split==14611 | year_split==14982 | year_split==15346 | year_split==15710 | year_split==16074 | year_split==16438 | year_split==16809 | year_split==17173 | year_split==17537 | year_split==17901 | year_split==18265 | year_split==18629 | year_split==18992

label define risk_seasonl 0 "NONE" 1 "hunt/hound" 2 "hunt/hound/snow" 3 "snow"

label values risk_season risk_seasonl

tab risk_season

tab risk_season if _d==1

tab cause_endpoint_enc risk_season if _d==1

*****PROTECTION PERIOD VARIABLE*****

stsplit treat_split, at(15795 16466 16526 16692 16914 17013 17236 17803 18020 18078 19018 19097) after(capture_date==1/1/1960)

***Generating liberalized killing treatment binary variable (1 if lib kill period)

gen lib_kill = 0

replace lib_kill = 1 if treat_split==15795 | treat_split==16526 | treat_split==16914 | treat_split==17236 ///

| treat_split==18020 | treat_split==19018

order lib_kill treat_split, after(cause_endpoint_agg)

tab lib_kill if _d==1

tab cause_endpoint2 lib_kill if _d==1

tab risk_season lib_kill if _d==1

*censoring all other created spells

replace event=0 if event==.

***SAVE DATASET***

save "Supp_Dataset_expanded.dta", replace

tab cause_endpoint_enc if _d==1

******************************************************************************

***JOINT ST COX MODELS FOR ALL CAUSE-SPECIFIC HAZARDS******************

******************************************************************************

*Checking stset by wolf_ID_exp (for multiple records)

stset date_endpoint, failure(event) exit(failure) origin(time capture_date) id(wolf_ID_exp)

stdes

stsum

******************************************************************************

***CAUSE-SPECIFIC HAZARD RATES FOR ALL CAUSES SIMULTANEOUSLY***

*Fit all in same model (basically same results as with separate PH models)

stcox 1.risk_season#2.cause_endpoint2 1.risk_season#3.cause_endpoint2 1.risk_season#4.cause_endpoint2 1.risk_season#5.cause_endpoint2 1.risk_season#6.cause_endpoint2 1.risk_season#7.cause_endpoint2 2.risk_season#2.cause_endpoint2 2.risk_season#3.cause_endpoint2 2.risk_season#4.cause_endpoint2 2.risk_season#5.cause_endpoint2 2.risk_season#6.cause_endpoint2 2.risk_season#7.cause_endpoint2 3.risk_season#2.cause_endpoint2 3.risk_season#3.cause_endpoint2 3.risk_season#4.cause_endpoint2 3.risk_season#5.cause_endpoint2 3.risk_season#6.cause_endpoint2 3.risk_season#7.cause_endpoint2, efron strata(cause_endpoint2 lib_kill) robust cluster(wolf_ID)

estimates store fullperiod_allLTFstrat

estat ic

/*checking assumptions*/

estat phtest, log detail

estat phtest, plot(1.risk_season#4.cause_endpoint2)

estat phtest, plot(2.risk_season#4.cause_endpoint2)

estat phtest, plot(1.risk_season#5.cause_endpoint2)

estat phtest, plot(2.risk_season#5.cause_endpoint2)

estat phtest, plot(3.risk_season#5.cause_endpoint2)

estat phtest, plot(2.risk_season#7.cause_endpoint2)

stphplot if cause_endpoint2==2, by(risk_season) nolnt

stphplot if cause_endpoint2==3, by(risk_season) nolnt

stphplot if cause_endpoint2==4, by(risk_season) nolnt

stphplot if cause_endpoint2==5, by(risk_season) nolnt

stphplot if cause_endpoint2==6, by(risk_season) nolnt

stphplot if cause_endpoint2==7, by(risk_season) nolnt

*WITH TVCs

stcox 1.risk_season#2.cause_endpoint2 1.risk_season#3.cause_endpoint2 1.risk_season#4.cause_endpoint2 1.risk_season#5.cause_endpoint2 1.risk_season#6.cause_endpoint2 1.risk_season#7.cause_endpoint2 2.risk_season#2.cause_endpoint2 2.risk_season#3.cause_endpoint2 2.risk_season#4.cause_endpoint2 2.risk_season#5.cause_endpoint2 2.risk_season#6.cause_endpoint2 2.risk_season#7.cause_endpoint2 3.risk_season#2.cause_endpoint2 3.risk_season#3.cause_endpoint2 3.risk_season#4.cause_endpoint2 3.risk_season#5.cause_endpoint2 3.risk_season#6.cause_endpoint2 3.risk_season#7.cause_endpoint2, tvc(1.risk_season#4.cause_endpoint2 1.risk_season#5.cause_endpoint2 2.risk_season#5.cause_endpoint2 2.risk_season#7.cause_endpoint2 3.risk_season#5.cause_endpoint2) texp(ln(_t)) efron strata(cause_endpoint2 lib_kill) robust cluster(wolf_ID)

estimates store fullperiod_allLTFTVCstrat

estat ic

stcox 1.risk_season#2.cause_endpoint2 1.risk_season#3.cause_endpoint2 ///

1.risk_season#4.cause_endpoint2 1.risk_season#5.cause_endpoint2 1.risk_season#6.cause_endpoint2 1.risk_season#7.cause_endpoint2 2.risk_season#2.cause_endpoint2 2.risk_season#3.cause_endpoint2 2.risk_season#4.cause_endpoint2 2.risk_season#5.cause_endpoint2 2.risk_season#6.cause_endpoint2 2.risk_season#7.cause_endpoint2 3.risk_season#2.cause_endpoint2 3.risk_season#3.cause_endpoint2 3.risk_season#4.cause_endpoint2 3.risk_season#5.cause_endpoint2 3.risk_season#6.cause_endpoint2 3.risk_season#7.cause_endpoint2, tvc(1.risk_season#5.cause_endpoint2 ///

2.risk_season#5.cause_endpoint2 2.risk_season#7.cause_endpoint2 3.risk_season#5.cause_endpoint2) texp(ln(_t)) efron strata(cause_endpoint2 lib_kill) robust cluster(wolf_ID)

estimates store fullperiod_allLTFTVCstratV2

estat ic

estout fullperiod_allLTFTVCstratV2, eform cells("b(star fmt(3)) ci_l ci_u" se(par fmt(2))) starlevels(* 0.10 ** .05 *** 0.01) stats(ll aic bic, labels("Log likelihood" "AIC" "BIC")) legend

*DIAGNOSTICS FOR STRATIFIED MODEL

stcox 1.risk_season#2.cause_endpoint2 1.risk_season#3.cause_endpoint2 1.risk_season#4.cause_endpoint2 1.risk_season#5.cause_endpoint2 1.risk_season#6.cause_endpoint2 1.risk_season#7.cause_endpoint2 2.risk_season#2.cause_endpoint2 2.risk_season#3.cause_endpoint2 2.risk_season#4.cause_endpoint2 2.risk_season#5.cause_endpoint2 2.risk_season#6.cause_endpoint2 2.risk_season#7.cause_endpoint2 3.risk_season#2.cause_endpoint2 3.risk_season#3.cause_endpoint2 3.risk_season#4.cause_endpoint2 3.risk_season#5.cause_endpoint2 3.risk_season#6.cause_endpoint2 3.risk_season#7.cause_endpoint2, efron strata(cause_endpoint2 lib_kill) robust cluster(wolf_ID)

estat phtest, plot(1.risk_season#2.cause_endpoint2) title("") ytitle("hunt/hound#collision") xtitle("days to end of monitoring") graphregion(color(white))

graph save "STCOX_JOINT_Schoenfeld_period1_COL", replace

estat phtest, plot(2.risk_season#2.cause_endpoint2) title("") ytitle("hunt/hound/snow#collision") xtitle("days to end of monitoring") graphregion(color(white))

graph save "STCOX_JOINT_Schoenfeld_period2_COL", replace

estat phtest, plot(3.risk_season#2.cause_endpoint2) title("") ytitle("snow#collision") xtitle("days to end of monitoring") graphregion(color(white))

graph save "STCOX_JOINT_Schoenfeld_period3_COL", replace

estat phtest, plot(1.risk_season#3.cause_endpoint2) title("") ytitle("hunt/hound#legal") xtitle("days to end of monitoring") graphregion(color(white))

graph save "STCOX_JOINT_Schoenfeld_period1_LEG", replace

estat phtest, plot(2.risk_season#3.cause_endpoint2) title("") ytitle("hunt/hound/snow#legal") xtitle("days to end of monitoring") graphregion(color(white))

graph save "STCOX_JOINT_Schoenfeld_period2_LEG", replace

estat phtest, plot(3.risk_season#3.cause_endpoint2) title("") ytitle("snow#legal") xtitle("days to end of monitoring") graphregion(color(white))

graph save "STCOX_JOINT_Schoenfeld_period3_LEG", replace

estat phtest, plot(1.risk_season#4.cause_endpoint2) title("") ytitle("hunt/hound#ltf") xtitle("days to end of monitoring") graphregion(color(white))

graph save "STCOX_JOINT_Schoenfeld_period1_LTF", replace

estat phtest, plot(2.risk_season#4.cause_endpoint2) title("") ytitle("hunt/hound/snow#ltf") xtitle("days to end of monitoring") graphregion(color(white))

graph save "STCOX_JOINT_Schoenfeld_period2_LTF", replace

estat phtest, plot(3.risk_season#4.cause_endpoint2) title("") ytitle("snow#ltf") xtitle("days to end of monitoring") graphregion(color(white))

graph save "STCOX_JOINT_Schoenfeld_period3_LTF", replace

estat phtest, plot(1.risk_season#5.cause_endpoint2) title("") ytitle("hunt/hound#nonhuman") xtitle("days to end of monitoring") graphregion(color(white))

graph save "STCOX_JOINT_Schoenfeld_period1_NON", replace

estat phtest, plot(2.risk_season#5.cause_endpoint2) title("") ytitle("hunt/hound/snow#nonhuman") xtitle("days to end of monitoring") graphregion(color(white))

graph save "STCOX_JOINT_Schoenfeld_period2_NON", replace

estat phtest, plot(3.risk_season#5.cause_endpoint2) title("") ytitle("snow#nonhuman") xtitle("days to end of monitoring") graphregion(color(white))

graph save "STCOX_JOINT_Schoenfeld_period3_NON", replace

estat phtest, plot(1.risk_season#6.cause_endpoint2) title("") ytitle("hunt/hound#poached") xtitle("days to end of monitoring") graphregion(color(white))

graph save "STCOX_JOINT_Schoenfeld_period1_POA", replace

estat phtest, plot(2.risk_season#6.cause_endpoint2) title("") ytitle("hunt/hound/snow#poached") xtitle("days to end of monitoring") graphregion(color(white))

graph save "STCOX_JOINT_Schoenfeld_period2_POA", replace

estat phtest, plot(3.risk_season#6.cause_endpoint2) title("") ytitle("snow#poached") xtitle("days to end of monitoring") graphregion(color(white))

graph save "STCOX_JOINT_Schoenfeld_period3_POA", replace

estat phtest, plot(1.risk_season#7.cause_endpoint2) title("") ytitle("hunt/hound#unknown") xtitle("days to end of monitoring") graphregion(color(white))

graph save "STCOX_JOINT_Schoenfeld_period1_UNK", replace

estat phtest, plot(2.risk_season#7.cause_endpoint2) title("") ytitle("hunt/hound/snow#unknown") xtitle("days to end of monitoring") graphregion(color(white))

graph save "STCOX_JOINT_Schoenfeld_period2_UNK", replace

estat phtest, plot(3.risk_season#7.cause_endpoint2) title("") ytitle("snow#unknown") xtitle("days to end of monitoring") graphregion(color(white))

graph save "STCOX_JOINT_Schoenfeld_period3_UNK", replace

graph combine "STCOX_JOINT_Schoenfeld_period1_COL" "STCOX_JOINT_Schoenfeld_period2_COL" "STCOX_JOINT_Schoenfeld_period3_COL" "STCOX_JOINT_Schoenfeld_period1_LEG" "STCOX_JOINT_Schoenfeld_period2_LEG" "STCOX_JOINT_Schoenfeld_period3_LEG" "STCOX_JOINT_Schoenfeld_period1_LTF" "STCOX_JOINT_Schoenfeld_period2_LTF" "STCOX_JOINT_Schoenfeld_period3_LTF" "STCOX_JOINT_Schoenfeld_period1_NON" "STCOX_JOINT_Schoenfeld_period2_NON" "STCOX_JOINT_Schoenfeld_period3_NON" "STCOX_JOINT_Schoenfeld_period1_POA" "STCOX_JOINT_Schoenfeld_period2_POA" "STCOX_JOINT_Schoenfeld_period3_POA" "STCOX_JOINT_Schoenfeld_period1_UNK" "STCOX_JOINT_Schoenfeld_period2_UNK" "STCOX_JOINT_Schoenfeld_period3_UNK", com graphregion(color(white)) saving("STCOX_JOINT_Schoenfeld_ALL", replace)

graph export "STCOX_JOINT_Schoenfeld_ALL.pdf", replace

*******stphplots********

stphplot if cause_endpoint2==2, by(risk_season) title("Collision") ytitle("−ln{−ln(survival)}") xtitle("days to end of monitoring") graphregion(color(white)) legend(order(1 "Baseline" 2 "hunt/hound" 3 "hunt/hound/snow" 4 "snow") region(lwidth(none))) nolnt

graph save "STCOX_JOINT_lnSurvYearPeriod_COL", replace

stphplot if cause_endpoint2==2, by(risk_season) title("Collision") ytitle("−ln{−ln(survival)}") xtitle("days to end of monitoring") graphregion(color(white)) legend(off) nolnt

graph save "STCOX_JOINT_lnSurvYearPeriod_COL_nolegend", replace

stphplot if cause_endpoint2==3, by(risk_season) title("Legal") ytitle("−ln{−ln(survival)}") xtitle("days to end of monitoring") graphregion(color(white)) legend(order(1 "Baseline" 2 "hunt/hound" 3 "hunt/hound/snow" 4 "snow") region(lwidth(none))) nolnt

graph save "STCOX_JOINT_lnSurvYearPeriod_LEG", replace

stphplot if cause_endpoint2==3, by(risk_season) title("Legal") ytitle("−ln{−ln(survival)}") xtitle("days to end of monitoring") graphregion(color(white)) legend(off) nolnt

graph save "STCOX_JOINT_lnSurvYearPeriod_LEG_nolegend", replace

stphplot if cause_endpoint2==4, by(risk_season) title("Lost-to-follow-up (LTF)") ytitle("−ln{−ln(survival)}") xtitle("days to end of monitoring") graphregion(color(white)) legend(order(1 "Baseline" 2 "hunt/hound" 3 "hunt/hound/snow" 4 "snow") region(lwidth(none))) nolnt

graph save "STCOX_JOINT_lnSurvYearPeriod_LTF", replace

stphplot if cause_endpoint2==4, by(risk_season) title("Lost-to-follow-up (LTF)") ytitle("−ln{−ln(survival)}") xtitle("days to end of monitoring") graphregion(color(white)) legend(off) nolnt

graph save "STCOX_JOINT_lnSurvYearPeriod_LTF_nolegend", replace

stphplot if cause_endpoint2==5, by(risk_season) title("Nonhuman (natural)") ytitle("−ln{−ln(survival)}") xtitle("days to end of monitoring") graphregion(color(white)) legend(order(1 "Baseline" 2 "hunt/hound" 3 "hunt/hound/snow" 4 "snow") region(lwidth(none))) nolnt

graph save "STCOX_JOINT_lnSurvYearPeriod_NON", replace

stphplot if cause_endpoint2==5, by(risk_season) title("Nonhuman (natural)") ytitle("−ln{−ln(survival)}") xtitle("days to end of monitoring") graphregion(color(white)) legend(off) nolnt

graph save "STCOX_JOINT_lnSurvYearPeriod_NON_nolegend", replace

stphplot if cause_endpoint2==6, by(risk_season) title("Reported poached") ytitle("−ln{−ln(survival)}") xtitle("days to end of monitoring") graphregion(color(white)) legend(order(1 "Baseline" 2 "hunt/hound" 3 "hunt/hound/snow" 4 "snow") region(lwidth(none))) nolnt

graph save "STCOX_JOINT_lnSurvYearPeriod_POA", replace

stphplot if cause_endpoint2==6, by(risk_season) title("Reported poached") ytitle("−ln{−ln(survival)}") xtitle("days to end of monitoring") graphregion(color(white)) legend(off) nolnt

graph save "STCOX_JOINT_lnSurvYearPeriod_POA_nolegend", replace

stphplot if cause_endpoint2==7, by(risk_season) title("Unknown cause") ytitle("−ln{−ln(survival)}") xtitle("days to end of monitoring") graphregion(color(white)) legend(order(1 "Baseline" 2 "hunt/hound" 3 "hunt/hound/snow" 4 "snow") region(lwidth(none))) nolnt

graph save "STCOX_JOINT_lnSurvYearPeriod_UNK", replace

stphplot if cause_endpoint2==7, by(risk_season) title("Unknown cause") ytitle("−ln{−ln(survival)}") xtitle("days to end of monitoring") graphregion(color(white)) legend(off) nolnt

graph save "STCOX_JOINT_lnSurvYearPeriod_UNK_nolegend", replace

graph combine "STCOX_JOINT_lnSurvYearPeriod_COL" "STCOX_JOINT_lnSurvYearPeriod_LEG" "STCOX_JOINT_lnSurvYearPeriod_LTF" "STCOX_JOINT_lnSurvYearPeriod_NON" "STCOX_JOINT_lnSurvYearPeriod_POA" "STCOX_JOINT_lnSurvYearPeriod_UNK", com graphregion(color(white)) saving("STCOX_JOINT_lnSurvYearPeriod_ByCauseEnd", replace)

graph export "STCOX_JOINT_lnSurvLibKill_ByCauseEnd.pdf", replace

*Goodness of fit**

predict cs, csnell

stset cs, id(wolf_ID_exp) failure(event)

sts generate H = na

line H cs cs, sort xlab(0 1 to 4) ylab(0 1 to 4) graphregion(color(white)) legend(region(lwidth(none)))

graph save "STCOX_JOINT_GoF", replace

graph export "STCOX_JOINT_GoF.pdf", replace

drop cs H

******************************************************************************

******************************************************************************

******************************************************************************

******************************************************************************

******************************************************************************

******************************************************************************

***CHECKING FOR CAUSE-SPECIFIC DIFFERENCES IN HAZARD & PH ASSUMPTION

******************************************************************************

**FOR THE DATASETS USED IN THIS .DO FILE**

use "Supp_Dataset.dta", replace

rename cause_endpoint2 old_endpoint

tab old_endpoint cause_endpoint

encode cause_endpoint, gen(cause_endpoint_enc)

label var cause_endpoint_enc "coded endpoint"

order cause_endpoint_enc, after(cause_endpoint)

tab cause_endpoint cause_endpoint_enc

stset date_endpoint, failure(cause_endpoint_enc==2 3 4 5 6 7) exit(failure) origin(time capture_date) id(wolf_ID)

*****TIME-SPLITTING for time-dependent variables and 'spells'*****

stsplit year_split, at(6946 7044 7120 7258 7310 7410 7486 7624 7674 7775 7851 7989 8038 8140 8216 8354 8402 8505 8581 8719 8773 8871 8947 9085 9137 9236 9312 9450 9501 9601 9677 9815 9865 9966 10042 10180 10229 10332 10408 10546 10600 10697 10773 10911 10964 11062 11138 11276 11328 11427 11503 11641 11692 11793 11869 12007 12056 12158 12234 12372 12420 12523 12599 12737 12791 12888 12964 13102 13155 13254 13330 13468 13519 13619 13695 13833 13883 13984 14060 14198 14247 14349 14425 14563 14611 14715 14791 14929 14982 15080 15156 15294 15346 15445 15521 15659 15710 15810 15886 16024 16074 16176 16252 16390 16438 16541 16617 16755 16809 16906 16982 17120 17173 17271 17347 17485 17537 17637 17713 17851 17901 18002 18078 18216 18265 18367 18443 18581 18629 18732 18808 18946 18992 19095 19098) after(capture_date==1/1/1960)

**Generating snow (1) time-dep binary variable

gen risk_season = 0

label var risk_season "intra-year periods"

replace risk_season = 1 if year_split==7120 | year_split==7486 | year_split==7851 | year_split==8216 | year_split==8581 | year_split==8947 | year_split==9312 | year_split==9677 | year_split==10042 | year_split==10408 | year_split==10773 | year_split==11138 | year_split==11503 | year_split==11869 | year_split==12234 | year_split==12599 | year_split==12964 | year_split==13330 | year_split==13695 | year_split==14060 | year_split==14425 | year_split==14791 | year_split==15156 | year_split==15521 | year_split==15886 | year_split==16252 | year_split==16617 | year_split==16982 | year_split==17347 | year_split==17713 | year_split==18078 | year_split==18443 | year_split==18808 | year_split==19098

replace risk_season = 2 if year_split==7258 | year_split==7624 | year_split==7989 | year_split==8354 | year_split==8719 | year_split==9085 | year_split==9450 | year_split==9815 | year_split==10180 | year_split==10546 | year_split==10911 | year_split==11276 | year_split==11641 | year_split==12007 | year_split==12372 | year_split==12737 | year_split==13102 | year_split==13468 | year_split==13833 | year_split==14198 | year_split==14563 | year_split==14929 | year_split==15294 | year_split==15659 | year_split==16024 | year_split==16390 | year_split==16755 | year_split==17120 | year_split==17485 | year_split==17851 | year_split==18216 | year_split==18581 | year_split==18946

replace risk_season = 3 if year_split==6946 | year_split==7310 | year_split==7674 | year_split==8038 | year_split==8402 | year_split==8773 | year_split==9137 | year_split==9501 | year_split==9865 | year_split==10229 | year_split==10600 | year_split==10964 | year_split==11328 | year_split==11692 | year_split==12056 | year_split==12420 | year_split==12791 | year_split==13155 | year_split==13519 | year_split==13883 | year_split==14247 | year_split==14611 | year_split==14982 | year_split==15346 | year_split==15710 | year_split==16074 | year_split==16438 | year_split==16809 | year_split==17173 | year_split==17537 | year_split==17901 | year_split==18265 | year_split==18629 | year_split==18992

label define risk_seasonl 0 "NONE" 1 "hunt/hound" 2 "hunt/hound/snow" 3 "snow"

label values risk_season risk_seasonl

tab risk_season

tab risk_season if _d==1

tab cause_endpoint_enc risk_season if _d==1

*****PROTECTION PERIOD VARIABLE*****

stsplit treat_split, at(15795 16466 16526 16692 16914 17013 17236 17803 18020 18078 19018 19097) after(capture_date==1/1/1960)

***Generating liberalized killing treatment binary variable (1 if lib kill period)

gen lib_kill = 0

replace lib_kill = 1 if treat_split==15795 | treat_split==16526 | treat_split==16914 | treat_split==17236 | treat_split==18020 | treat_split==19018

order lib_kill treat_split, after(cause_endpoint_agg)

label define lib_killl 0 "Strict protections" 1 "Reduced protections"

label values lib_kill lib_killl

tab lib_kill if _d==1

tab cause_endpoint_enc lib_kill if _d==1

sort capture_date date_endpoint wolf_ID

gen obs_order = _n

order obs_order, before(wolf_ID)

save "Supp_Dataset_split.dta", replace

******************************************************************************

****ST COX FOR CAUSE-SPECIFIC HAZARDS************************************

******************************************************************************

use "Supp_Dataset_split.dta", replace

**COLLISION**

stset date_endpoint, failure(cause_endpoint_enc==2) exit(failure) origin(time capture_date) id(wolf_ID)

stcox i.risk_season, strata(lib_kill) efron robust cluster(wolf_ID)

predict Ch_col_0, basec

gsort _t -_d

by _t: replace Ch_col_0 = . if _n > 1

gen Ch_col_1 = Ch_col_0*exp(_b[1.risk_season])

gen Ch_col_2 = Ch_col_0*exp(_b[2.risk_season])

gen Ch_col_3 = Ch_col_0*exp(_b[3.risk_season])

twoway line Ch_col_* _t if _d==1 & lib_kill==0, connect(stepstair stepstair stepstair stepstair) sort lpattern(solid dash_dot dash longdash) lcolor(navy orange maroon green) graphregion(color(white)) legend(off)

graph save "STCOX_CUMHAZ_COL_STR", replace

twoway line Ch_col_* _t if _d==1 & lib_kill==1, connect(stepstair stepstair stepstair stepstair) sort lpattern(solid dash_dot dash longdash) lcolor(navy orange maroon green) graphregion(color(white)) legend(off)

graph save "STCOX_CUMHAZ_COL_RED", replace

graph combine "STCOX_CUMHAZ_COL_STR" "STCOX_CUMHAZ_COL_RED", graphregion(color(white)) com saving("STCOX_CUMHAZ_COL_COMB", replace)

stset date_endpoint, failure(cause_endpoint_enc==2) exit(failure) origin(time capture_date) id(wolf_ID)

stcox i.risk_season, strata(lib_kill) efron robust cluster(wolf_ID)

*predicting HR for CIFs later

predict h_col_0, basehc

gsort _t -_d

by _t: replace h_col_0 = . if _n > 1

gen h_col_1 = h_col_0*exp(_b[1.risk_season])

gen h_col_2 = h_col_0*exp(_b[2.risk_season])

gen h_col_3 = h_col_0*exp(_b[3.risk_season])

predict cs, csnell

stset cs, id(wolf_ID) failure(cause_endpoint_enc==2)

sts generate H = na

line H cs cs, sort xlab(0 1 to 4) ylab(0 1 to 4)

drop cs H

**LEGAL**

stset date_endpoint, failure(cause_endpoint_enc==3) exit(failure) origin(time capture_date) id(wolf_ID)

stcox i.risk_season, strata(lib_kill) efron robust cluster(wolf_ID)

predict Ch_leg_0, basec

gsort _t -_d

by _t: replace Ch_leg_0 = . if _n > 1

gen Ch_leg_1 = Ch_leg_0*exp(_b[1.risk_season])

gen Ch_leg_2 = Ch_leg_0*exp(_b[2.risk_season])

gen Ch_leg_3 = Ch_leg_0*exp(_b[3.risk_season])

twoway line Ch_leg_* _t if _d==1 & lib_kill==0, connect(stepstair stepstair stepstair stepstair) sort lpattern(solid dash_dot dash longdash) lcolor(navy orange maroon green) graphregion(color(white)) legend(off)

graph save "STCOX_CUMHAZ_LEG_STR", replace

twoway line Ch_leg_* _t if _d==1 & lib_kill==1, connect(stepstair stepstair stepstair stepstair) sort lpattern(solid dash_dot dash longdash) lcolor(navy orange maroon green) graphregion(color(white)) legend(off)

graph save "STCOX_CUMHAZ_LEG_RED", replace

graph combine "STCOX_CUMHAZ_LEG_STR" "STCOX_CUMHAZ_LEG_RED", graphregion(color(white)) com saving("STCOX_CUMHAZ_LEG_COMB", replace)

*predicting HR for CIFs later

predict h_leg_0, basehc

gsort _t -_d

by _t: replace h_leg_0 = . if _n > 1

gen h_leg_1 = h_leg_0*exp(_b[1.risk_season])

gen h_leg_2 = h_leg_0*exp(_b[2.risk_season])

gen h_leg_3 = h_leg_0*exp(_b[3.risk_season])

predict cs, csnell

stset cs, id(wolf_ID) failure(cause_endpoint_enc==3)

sts generate H = na

line H cs cs, sort xlab(0 1 to 4) ylab(0 1 to 4)

drop cs H

**LTF**

stset date_endpoint, failure(cause_endpoint_enc==4) exit(failure) origin(time capture_date) id(wolf_ID)

stcox i.risk_season, strata(lib_kill) efron robust cluster(wolf_ID)

predict Ch_ltf_0, basec

gsort _t -_d

by _t: replace Ch_ltf_0 = . if _n > 1

gen Ch_ltf_1 = Ch_ltf_0*exp(_b[1.risk_season])

gen Ch_ltf_2 = Ch_ltf_0*exp(_b[2.risk_season])

gen Ch_ltf_3 = Ch_ltf_0*exp(_b[3.risk_season])

twoway line Ch_ltf_* _t if _d==1 & lib_kill==0, connect(stepstair stepstair stepstair stepstair) sort lpattern(solid dash_dot dash longdash) lcolor(navy orange maroon green) graphregion(color(white)) legend(off)

graph save "STCOX_CUMHAZ_LTF_STR", replace

twoway line Ch_ltf_* _t if _d==1 & lib_kill==1, connect(stepstair stepstair stepstair stepstair) sort lpattern(solid dash_dot dash longdash) lcolor(navy orange maroon green) graphregion(color(white)) legend(off)

graph save "STCOX_CUMHAZ_LTF_RED", replace

graph combine "STCOX_CUMHAZ_LTF_STR" "STCOX_CUMHAZ_LTF_RED", graphregion(color(white)) com saving("STCOX_CUMHAZ_LTF_COMB", replace)

*predicting HR for CIFs later

predict h_ltf_0, basehc

gsort _t -_d

by _t: replace h_ltf_0 = . if _n > 1

gen h_ltf_1 = h_ltf_0*exp(_b[1.risk_season])

gen h_ltf_2 = h_ltf_0*exp(_b[2.risk_season])

gen h_ltf_3 = h_ltf_0*exp(_b[3.risk_season])

predict cs, csnell

stset cs, id(wolf_ID) failure(cause_endpoint_enc==4)

sts generate H = na

line H cs cs, sort xlab(0 1 to 4) ylab(0 1 to 4)

drop cs H

**POACHED**

stset date_endpoint, failure(cause_endpoint_enc==6) exit(failure) origin(time capture_date) id(wolf_ID)

stcox i.risk_season, strata(lib_kill) efron robust cluster(wolf_ID)

predict Ch_poa_0, basec

gsort _t -_d

by _t: replace Ch_poa_0 = . if _n > 1

gen Ch_poa_1 = Ch_poa_0*exp(_b[1.risk_season])

gen Ch_poa_2 = Ch_poa_0*exp(_b[2.risk_season])

gen Ch_poa_3 = Ch_poa_0*exp(_b[3.risk_season])

twoway line Ch_poa_* _t if _d==1 & lib_kill==0, connect(stepstair stepstair stepstair stepstair) sort lpattern(solid dash_dot dash longdash) lcolor(navy orange maroon green) graphregion(color(white)) legend(off)

graph save "STCOX_CUMHAZ_POA_STR", replace

twoway line Ch_poa_* _t if _d==1 & lib_kill==1, connect(stepstair stepstair stepstair stepstair) sort lpattern(solid dash_dot dash longdash) lcolor(navy orange maroon green) graphregion(color(white)) legend(off)

graph save "STCOX_CUMHAZ_POA_RED", replace

graph combine "STCOX_CUMHAZ_POA_STR" "STCOX_CUMHAZ_POA_RED", graphregion(color(white)) com saving("STCOX_CUMHAZ_POA_COMB", replace)

*predicting HR for CIFs later

predict h_poa_0, basehc

gsort _t -_d

by _t: replace h_poa_0 = . if _n > 1

gen h_poa_1 = h_poa_0*exp(_b[1.risk_season])

gen h_poa_2 = h_poa_0*exp(_b[2.risk_season])

gen h_poa_3 = h_poa_0*exp(_b[3.risk_season])

predict cs, csnell

stset cs, id(wolf_ID) failure(cause_endpoint_enc==6)

sts generate H = na

line H cs cs, sort xlab(0 1 to 4) ylab(0 1 to 4)

drop cs H

**UNKNOWN**

stset date_endpoint, failure(cause_endpoint_enc==7) exit(failure) origin(time capture_date) id(wolf_ID)

stcox i.risk_season, strata(lib_kill) efron robust cluster(wolf_ID)

predict Ch_unk_0, basec

gsort _t -_d

by _t: replace Ch_unk_0 = . if _n > 1

gen Ch_unk_1 = Ch_unk_0*exp(_b[1.risk_season])

gen Ch_unk_2 = Ch_unk_0*exp(_b[2.risk_season])

gen Ch_unk_3 = Ch_unk_0*exp(_b[3.risk_season])

twoway line Ch_unk_* _t if _d==1 & lib_kill==0, connect(stepstair stepstair stepstair stepstair) sort lpattern(solid dash_dot dash longdash) lcolor(navy orange maroon green) graphregion(color(white)) legend(off)

graph save "STCOX_CUMHAZ_UNK_STR", replace

twoway line Ch_unk_* _t if _d==1 & lib_kill==1, connect(stepstair stepstair stepstair stepstair) sort lpattern(solid dash_dot dash longdash) lcolor(navy orange maroon green) graphregion(color(white)) legend(off)

graph save "STCOX_CUMHAZ_UNK_RED", replace

graph combine "STCOX_CUMHAZ_UNK_STR" "STCOX_CUMHAZ_UNK_RED", graphregion(color(white)) com saving("STCOX_CUMHAZ_UNK_COMB", replace)

*predicting HR for CIFs later

predict h_unk_0, basehc

gsort _t -_d

by _t: replace h_unk_0 = . if _n > 1

gen h_unk_1 = h_unk_0*exp(_b[1.risk_season])

gen h_unk_2 = h_unk_0*exp(_b[2.risk_season])

gen h_unk_3 = h_unk_0*exp(_b[3.risk_season])

predict cs, csnell

stset cs, id(wolf_ID) failure(cause_endpoint_enc==7)

sts generate H = na

line H cs cs, sort xlab(0 1 to 4) ylab(0 1 to 4)

drop cs H

**NONHUMAN**

stset date_endpoint, failure(cause_endpoint_enc==5) exit(failure) origin(time capture_date) id(wolf_ID)

stcox i.risk_season, strata(lib_kill) efron robust cluster(wolf_ID)

stcox i.risk_season, tvc(i.risk_season) texp(ln(_t)) strata(lib_kill) efron robust cluster(wolf_ID)

*for tvcs without the command (to use for predict command below)

stsplit, at(failures)

gen lnt = ln(_t)

stcox i.risk_season 1.risk_season#c.lnt 2.risk_season#c.lnt 3.risk_season#c.lnt, strata(lib_kill) efron robust cluster(wolf_ID)

stcox i.risk_season 1.risk_season#c.lnt 2.risk_season#c.lnt 3.risk_season#c.lnt, strata(lib_kill) efron robust cluster(wolf_ID) nohr

predict Ch_non_0, basec

gsort _t -_d

by _t: replace Ch_non_0 = . if _n > 1

gen Ch_non_1 = Ch_non_0*exp(_b[1.risk_season]+(-.9459155*lnt))

gen Ch_non_2 = Ch_non_0*exp(_b[2.risk_season]+(-.8267926*lnt))

gen Ch_non_3 = Ch_non_0*exp(_b[3.risk_season]+(-.8997016*lnt))

twoway line Ch_non_* _t if _d==1 & lib_kill==0, connect(stepstair stepstair stepstair stepstair) sort lpattern(solid dash_dot dash longdash) lcolor(navy orange maroon green) graphregion(color(white)) legend(off)

graph save "STCOX_CUMHAZ_NON_STR", replace

twoway line Ch_non_* _t if _d==1 & lib_kill==1, connect(stepstair stepstair stepstair stepstair) sort lpattern(solid dash_dot dash longdash) lcolor(navy orange maroon green) graphregion(color(white)) legend(off)

graph save "STCOX_CUMHAZ_NON_RED", replace

graph combine "STCOX_CUMHAZ_NON_STR" "STCOX_CUMHAZ_NON_RED", com saving("STCOX_CUMHAZ_NON_COMB", replace)

*predicting HR for CIFs later

predict h_non_0, basehc

gsort _t -_d

by _t: replace h_non_0 = . if _n > 1

gen h_non_1 = h_non_0*exp(_b[1.risk_season]+(-.9459155*lnt))

gen h_non_2 = h_non_0*exp(_b[2.risk_season]+(-.8267926*lnt))

gen h_non_3 = h_non_0*exp(_b[3.risk_season]+(-.8997016*lnt))

predict cs, csnell

stset cs, id(wolf_ID) failure(cause_endpoint_enc==5)

sts generate H = na

line H cs cs, sort xlab(0 1 to 4) ylab(0 1 to 4)

drop cs H

stset date_endpoint, failure(cause_endpoint_enc==2 3 4 5 6 7) exit(failure) origin(time capture_date) id(wolf_ID)

twoway line Ch_ltf_0 Ch_ltf_1 Ch_poa_0 Ch_poa_1 Ch_leg_0 Ch_leg_1 Ch_non_0 Ch_non_1 _t if _t<=2000 & _d==1 & lib_kill==0, connect(stepstair stepstair stepstair stepstair stepstair stepstair stepstair stepstair)sort lpattern(solid solid longdash longdash shortdash_dot shortdash_dot dot dot) lcolor(black gray black gray black gray black gray) ytitle("Cumulative hazard") xtitle("Analysis time (t, days)") graphregion(color(white)) legend(off)

graph save "STCOX_Ch_LTF_POA_LEG_NON_COMB_1_BG_t", replace

twoway line Ch_ltf_0 Ch_ltf_2 Ch_poa_0 Ch_poa_2 Ch_leg_0 Ch_leg_2 Ch_non_0 Ch_non_2 _t if _t<=2000 & _d==1 & lib_kill==0, connect(stepstair stepstair stepstair stepstair stepstair stepstair stepstair stepstair)sort lpattern(solid solid longdash longdash shortdash_dot shortdash_dot dot dot) lcolor(black gray black gray black gray black gray) ytitle("Cumulative hazard") xtitle("Analysis time (t, days)") graphregion(color(white)) legend(off)

graph save "STCOX_Ch_LTF_POA_LEG_NON_COMB_2_BG_t", replace

twoway line Ch_ltf_0 Ch_ltf_3 Ch_poa_0 Ch_poa_3 Ch_leg_0 Ch_leg_3 Ch_non_0 Ch_non_3 _t if _t<=2000 & _d==1 & lib_kill==0, connect(stepstair stepstair stepstair stepstair stepstair stepstair stepstair stepstair)sort lpattern(solid solid longdash longdash shortdash_dot shortdash_dot dot dot) lcolor(black gray black gray black gray black gray) ytitle("Cumulative hazard") xtitle("Analysis time (t, days)") graphregion(color(white)) legend(off)

graph save "STCOX_Ch_LTF_POA_LEG_NON_COMB_3_BG_t", replace

twoway line Ch_non_* _t

gen Ch_hts_poa_ltf = Ch_poa_2/Ch_ltf_2

sum Ch_hts_poa_ltf

twoway line Ch_hts_poa_ltf _t if _t<=2000 & _d==1 & lib_kill==0, connect(stepstair) sort lpattern(solid) lcolor(black) ytitle("Cumulative hazard") xtitle("Analysis time (t, days)") graphregion(color(white)) legend(off)

drop Ch_hts_poa_ltf

gen Ch_hts_non_ltf = Ch_non_2/Ch_ltf_2

sum Ch_hts_non_ltf if _d==1 & lib_kill==0

twoway line Ch_hts_non_ltf _t if _t<=2000 & _d==1 & lib_kill==0, connect(stepstair) sort lpattern(solid) lcolor(black) ytitle("Cumulative hazard") xtitle("Analysis time (t, days)") graphregion(color(white)) legend(off)

gen Ch_s_non_ltf = Ch_non_3/Ch_ltf_3

sum Ch_s_non_ltf if _d==1 & lib_kill==0 & _t>=100

twoway line Ch_s_non_ltf _t if _t<=2000 & _d==1 & lib_kill==0, connect(stepstair) sort lpattern(solid) lcolor(black) ytitle("Cumulative hazard") xtitle("Analysis time (t, days)") graphregion(color(white)) legend(off)

gen Ch_s_poa_ltf = Ch_poa_3/Ch_ltf_3

sum Ch_s_poa_ltf if _d==1 & lib_kill==0 & _t>=100

twoway line Ch_s_poa_ltf _t if _t<=2000 & _d==1 & lib_kill==0, connect(stepstair) sort lpattern(solid) lcolor(black) ytitle("Cumulative hazard") xtitle("Analysis time (t, days)") graphregion(color(white)) legend(off)

gen Ch_s_non_poa = Ch_non_3/Ch_poa_3

sum Ch_s_non_poa if _d==1 & lib_kill==0 & _t>=100

twoway line Ch_s_non_poa _t if _t<=2000 & _d==1 & lib_kill==0, connect(stepstair) sort lpattern(solid) lcolor(black) ytitle("Cumulative hazard") xtitle("Analysis time (t, days)") graphregion(color(white)) legend(off)

******************************************************************************

**CREATING CIFs WITH ABOVE HAZARD CONTRIBUTIONS

stset date_endpoint, failure(cause_endpoint_enc==2 3 4 5 6 7) exit(failure) origin(time capture_date) id(wolf_ID)

drop if missing(h_col_0) & missing(h_leg_0) & missing(h_ltf_0) & missing(h_non_0) & missing(h_poa_0) & missing(h_unk_0)

replace h_col_0=0 if missing(h_col_0)

replace h_col_1=0 if missing(h_col_1)

replace h_col_2=0 if missing(h_col_2)

replace h_col_3=0 if missing(h_col_3)

replace h_leg_0=0 if missing(h_leg_0)

replace h_leg_1=0 if missing(h_leg_1)

replace h_leg_2=0 if missing(h_leg_2)

replace h_leg_3=0 if missing(h_leg_3)

replace h_ltf_0=0 if missing(h_ltf_0)

replace h_ltf_1=0 if missing(h_ltf_1)

replace h_ltf_2=0 if missing(h_ltf_2)

replace h_ltf_3=0 if missing(h_ltf_3)

replace h_non_0=0 if missing(h_non_0)

replace h_non_1=0 if missing(h_non_1)

replace h_non_2=0 if missing(h_non_2)

replace h_non_3=0 if missing(h_non_3)

replace h_poa_0=0 if missing(h_poa_0)

replace h_poa_1=0 if missing(h_poa_1)

replace h_poa_2=0 if missing(h_poa_2)

replace h_poa_3=0 if missing(h_poa_3)

replace h_unk_0=0 if missing(h_unk_0)

replace h_unk_1=0 if missing(h_unk_1)

replace h_unk_2=0 if missing(h_unk_2)

replace h_unk_3=0 if missing(h_unk_3)

**calculating event-free survivor functions

sort _t

gen S_0 = exp(sum(log(1- h_col_0 - h_leg_0 - h_ltf_0 - h_non_0 - h_poa_0 - h_unk_0))) if _d==1

gen S_1 = exp(sum(log(1- h_col_1 - h_leg_1 - h_ltf_1 - h_non_1 - h_poa_1 - h_unk_1))) if _d==1

gen S_2 = exp(sum(log(1- h_col_2 - h_leg_2 - h_ltf_2 - h_non_2 - h_poa_2 - h_unk_2))) if _d==1

gen S_3 = exp(sum(log(1- h_col_3 - h_leg_3 - h_ltf_3 - h_non_3 - h_poa_3 - h_unk_3))) if _d==1

twoway line S_* _t if t<=2000, connect(stepstair stepstair stepstair stepstair) sort lpattern(solid dash_dot dash longdash) lcolor(navy orange maroon green) graphregion(color(white)) legend(off)

graph save "STCOX_CIF_ALL", replace

twoway line S_* _t if _t<=300 & lib_kill==0, connect(stepstair stepstair stepstair stepstair) sort lpattern(solid dash_dot dash longdash) lcolor(navy orange maroon green) graphregion(color(white)) legend(off)

graph save "STCOX_CIF_ALL_STR", replace

twoway line S_* _t if _t<=500 & lib_kill==1, connect(stepstair stepstair stepstair stepstair) sort lpattern(solid dash_dot dash longdash) lcolor(navy orange maroon green) graphregion(color(white)) legend(off)

graph save "STCOX_CIF_ALL_RED", replace

graph combine "STCOX_CIF_ALL_STR" "STCOX_CIF_ALL_RED", graphregion(color(white)) com saving("STCOX_CIF_ALL_COMB", replace)

*calculating CIFs

*colissions

gen cif_col_0 = sum(S_0[_n-1]*h_col_0)

gen cif_col_1 = sum(S_1[_n-1]*h_col_1)

gen cif_col_2 = sum(S_2[_n-1]*h_col_2)

gen cif_col_3 = sum(S_3[_n-1]*h_col_3)

twoway line cif_col_* _t if _t<=300, connect(stepstair stepstair stepstair stepstair) sort lpattern(solid dash_dot dash longdash) lcolor(navy orange maroon green) graphregion(color(white)) legend(off)

graph save "STCOX_CIF_COL", replace

twoway line cif_col_* _t if _t<=300 & lib_kill==0, connect(stepstair stepstair stepstair stepstair) sort lpattern(solid dash_dot dash longdash) lcolor(navy orange maroon green) graphregion(color(white)) legend(off)

graph save "STCOX_CIF_COL_STR", replace

twoway line cif_col_* _t if _t<=300 & lib_kill==1, connect(stepstair stepstair stepstair stepstair) sort lpattern(solid dash_dot dash longdash) lcolor(navy orange maroon green) graphregion(color(white)) legend(off)

graph save "STCOX_CIF_COL_RED", replace

graph combine "STCOX_CIF_COL_STR" "STCOX_CIF_COL_RED", graphregion(color(white)) com saving("STCOX_CIF_COL_COMB", replace)

*legal

gen cif_leg_0 = sum(S_0[_n-1]*h_leg_0)

gen cif_leg_1 = sum(S_1[_n-1]*h_leg_1)

gen cif_leg_2 = sum(S_2[_n-1]*h_leg_2)

gen cif_leg_3 = sum(S_3[_n-1]*h_leg_3)

twoway line cif_leg_* _t if _t<=300, connect(stepstair stepstair stepstair stepstair) sort lpattern(solid dash_dot dash longdash) lcolor(navy orange maroon green) graphregion(color(white)) legend(off)

graph save "STCOX_CIF_LEG", replace

twoway line cif_leg_* _t if _t<=300 & lib_kill==0, connect(stepstair stepstair stepstair stepstair) sort lpattern(solid dash_dot dash longdash) lcolor(navy orange maroon green) graphregion(color(white)) legend(off)

graph save "STCOX_CIF_LEG_STR", replace

twoway line cif_leg_* _t if _t<=300 & lib_kill==1, connect(stepstair stepstair stepstair stepstair) sort lpattern(solid dash_dot dash longdash) lcolor(navy orange maroon green) graphregion(color(white)) legend(off)

graph save "STCOX_CIF_LEG_RED", replace

graph combine "STCOX_CIF_LEG_STR" "STCOX_CIF_LEG_RED", graphregion(color(white)) com saving("STCOX_CIF_LEG_COMB", replace)

*ltf

gen cif_ltf_0 = sum(S_0[_n-1]*h_ltf_0)

gen cif_ltf_1 = sum(S_1[_n-1]*h_ltf_1)

gen cif_ltf_2 = sum(S_2[_n-1]*h_ltf_2)

gen cif_ltf_3 = sum(S_3[_n-1]*h_ltf_3)

twoway line cif_ltf_* _t if _t<=300, connect(stepstair stepstair stepstair stepstair) sort lpattern(solid dash_dot dash longdash) lcolor(navy orange maroon green) graphregion(color(white)) legend(off)

graph save "STCOX_CIF_LTF", replace

twoway line cif_ltf_* _t if _t<=300 & lib_kill==0, connect(stepstair stepstair stepstair stepstair) sort lpattern(solid dash_dot dash longdash) lcolor(navy orange maroon green) graphregion(color(white)) legend(off)

graph save "STCOX_CIF_LTF_STR", replace

twoway line cif_ltf_* _t if _t<=300 & lib_kill==1, connect(stepstair stepstair stepstair stepstair) sort lpattern(solid dash_dot dash longdash) lcolor(navy orange maroon green) graphregion(color(white)) legend(off)

graph save "STCOX_CIF_LTF_RED", replace

graph combine "STCOX_CIF_LTF_STR" "STCOX_CIF_LTF_RED", graphregion(color(white)) com saving("STCOX_CIF_LTF_COMB", replace)

*nonhuman

gen cif_non_0 = sum(S_0[_n-1]*h_non_0)

gen cif_non_1 = sum(S_1[_n-1]*h_non_1)

gen cif_non_2 = sum(S_2[_n-1]*h_non_2)

gen cif_non_3 = sum(S_3[_n-1]*h_non_3)

twoway line cif_non_* _t if _t<=300, connect(stepstair stepstair stepstair stepstair) sort lpattern(solid dash_dot dash longdash) lcolor(navy orange maroon green) graphregion(color(white)) legend(off)

graph save "STCOX_CIF_NON", replace

twoway line cif_non_* _t if _t<=300 & lib_kill==0, connect(stepstair stepstair stepstair stepstair) sort lpattern(solid dash_dot dash longdash) lcolor(navy orange maroon green) graphregion(color(white)) legend(off)

graph save "STCOX_CIF_NON_STR", replace

twoway line cif_non_* _t if _t<=300 & lib_kill==1, connect(stepstair stepstair stepstair stepstair) sort lpattern(solid dash_dot dash longdash) lcolor(navy orange maroon green) graphregion(color(white)) legend(off)

graph save "STCOX_CIF_NON_RED", replace

graph combine "STCOX_CIF_NON_STR" "STCOX_CIF_NON_RED", com saving("STCOX_CIF_NON_COMB", replace)

*poached

gen cif_poa_0 = sum(S_0[_n-1]*h_poa_0)

gen cif_poa_1 = sum(S_1[_n-1]*h_poa_1)

gen cif_poa_2 = sum(S_2[_n-1]*h_poa_2)

gen cif_poa_3 = sum(S_3[_n-1]*h_poa_3)

twoway line cif_poa_* _t if _t<=300, connect(stepstair stepstair stepstair stepstair) sort lpattern(solid dash_dot dash longdash) lcolor(navy orange maroon green) graphregion(color(white)) legend(off)

graph save "STCOX_CIF_POA", replace

twoway line cif_poa_* _t if _t<=300 & lib_kill==0, connect(stepstair stepstair stepstair stepstair) sort lpattern(solid dash_dot dash longdash) lcolor(navy orange maroon green) graphregion(color(white)) legend(off)

graph save "STCOX_CIF_POA_STR", replace

twoway line cif_poa_* _t if _t<=300 & lib_kill==1, connect(stepstair stepstair stepstair stepstair) sort lpattern(solid dash_dot dash longdash) lcolor(navy orange maroon green) graphregion(color(white)) legend(off)

graph save "STCOX_CIF_POA_RED", replace

graph combine "STCOX_CIF_POA_STR" "STCOX_CIF_POA_RED", graphregion(color(white)) com saving("STCOX_CIF_POA_COMB", replace)

*unknown

gen cif_unk_0 = sum(S_0[_n-1]*h_unk_0)

gen cif_unk_1 = sum(S_1[_n-1]*h_unk_1)

gen cif_unk_2 = sum(S_2[_n-1]*h_unk_2)

gen cif_unk_3 = sum(S_3[_n-1]*h_unk_3)

twoway line cif_unk_* _t if _t<=300, connect(stepstair stepstair stepstair stepstair) sort lpattern(solid dash_dot dash longdash) lcolor(navy orange maroon green) graphregion(color(white)) legend(off)

graph save "STCOX_CIF_UNK", replace

twoway line cif_unk_* _t if _t<=300 & lib_kill==0, connect(stepstair stepstair stepstair stepstair) sort lpattern(solid dash_dot dash longdash) lcolor(navy orange maroon green) graphregion(color(white)) legend(off)

graph save "STCOX_CIF_UNK_STR", replace

twoway line cif_unk_* _t if _t<=300 & lib_kill==1, connect(stepstair stepstair stepstair stepstair) sort lpattern(solid dash_dot dash longdash) lcolor(navy orange maroon green) graphregion(color(white)) legend(off)

graph save "STCOX_CIF_UNK_RED", replace

graph combine "STCOX_CIF_UNK_STR" "STCOX_CIF_UNK_RED", graphregion(color(white)) com saving("STCOX_CIF_UNK_COMB", replace)

twoway line cif_ltf_* cif_poa_* cif_leg_* _t, connect(stepstair stepstair stepstair stepstair stepstair stepstair stepstair stepstair stepstair stepstair stepstair stepstair) sort lpattern(solid solid solid solid longdash longdash longdash longdash shortdash_dot shortdash_dot shortdash_dot shortdash_dot) lcolor(navy orange maroon green navy orange maroon green navy orange maroon green) graphregion(color(white)) legend(off)

graph save "STCOX_CIF_LTF_POA_LEG_COMB", replace

twoway line cif_ltf_* cif_poa_* cif_non_* _t if _t<=200, connect(stepstair stepstair stepstair stepstair stepstair stepstair stepstair stepstair stepstair stepstair stepstair stepstair) sort lpattern(solid solid solid solid longdash longdash longdash longdash shortdash_dot shortdash_dot shortdash_dot shortdash_dot) lcolor(navy orange maroon green navy orange maroon green navy orange maroon green) graphregion(color(white)) legend(off)

graph save "STCOX_CIF_LTF_POA_NON_COMB", replace

twoway line cif_ltf_0 cif_ltf_1 cif_poa_0 cif_poa_1 cif_leg_0 cif_leg_1 _t if _t<=200, connect(stepstair stepstair stepstair stepstair stepstair stepstair)sort lpattern(solid solid longdash longdash shortdash_dot shortdash_dot) lcolor(navy orange navy orange avy orange ) graphregion(color(white)) legend(off)

graph save "STCOX_CIF_LTF_POA_LEG_COMB", replace

twoway line cif_ltf_0 cif_ltf_1 cif_poa_0 cif_poa_1 cif_leg_0 cif_leg_1 cif_non_0 cif_non_1 _t if _t<=200, connect(stepstair stepstair stepstair stepstair stepstair stepstair stepstair stepstair)sort lpattern(solid solid longdash longdash shortdash_dot shortdash_dot dot dot) lcolor(navy orange navy orange navy orange navy orange) graphregion(color(white)) legend(off)

graph save "STCOX_CIF_LTF_POA_LEG_NON_COMB_1", replace

twoway line cif_ltf_0 cif_ltf_2 cif_poa_0 cif_poa_2 cif_leg_0 cif_leg_2 cif_non_0 cif_non_2 _t if _t<=200, connect(stepstair stepstair stepstair stepstair stepstair stepstair stepstair stepstair)sort lpattern(solid solid longdash longdash shortdash_dot shortdash_dot dot dot) lcolor(navy maroon navy maroon navy maroon navy maroon) graphregion(color(white)) legend(off)

graph save "STCOX_CIF_LTF_POA_LEG_NON_COMB_2", replace

twoway line cif_ltf_0 cif_ltf_3 cif_poa_0 cif_poa_3 cif_leg_0 cif_leg_3 cif_non_0 cif_non_3 _t if _t<=200, connect(stepstair stepstair stepstair stepstair stepstair stepstair stepstair stepstair)sort lpattern(solid solid longdash longdash shortdash_dot shortdash_dot dot dot) lcolor(navy green navy green navy green navy green) graphregion(color(white)) legend(off)

graph save "STCOX_CIF_LTF_POA_LEG_NON_COMB_3", replace

**BLACK/GRAY FIGURES**

twoway line cif_ltf_0 cif_ltf_1 cif_poa_0 cif_poa_1 cif_leg_0 cif_leg_1 cif_non_0 cif_non_1 _t if _t<=2000, connect(stepstair stepstair stepstair stepstair stepstair stepstair stepstair stepstair)sort lpattern(solid solid longdash longdash shortdash_dot shortdash_dot dot dot) lcolor(black gray black gray black gray black gray) ytitle("Cumulative incidence") xtitle("Analysis time (t, days)") graphregion(color(white)) legend(off)

graph save "STCOX_CIF_LTF_POA_LEG_NON_COMB_1_BG", replace

twoway line cif_ltf_0 cif_ltf_2 cif_poa_0 cif_poa_2 cif_leg_0 cif_leg_2 cif_non_0 cif_non_2 _t if _t<=2000, connect(stepstair stepstair stepstair stepstair stepstair stepstair stepstair stepstair)sort lpattern(solid solid longdash longdash shortdash_dot shortdash_dot dot dot) lcolor(black gray black gray black gray black gray) ytitle("Cumulative incidence") xtitle("Analysis time (t, days)") graphregion(color(white)) legend(off)

graph save "STCOX_CIF_LTF_POA_LEG_NON_COMB_2_BG", replace

twoway line cif_ltf_0 cif_ltf_3 cif_poa_0 cif_poa_3 cif_leg_0 cif_leg_3 cif_non_0 cif_non_3 _t if _t<=2000, connect(stepstair stepstair stepstair stepstair stepstair stepstair stepstair stepstair)sort lpattern(solid solid longdash longdash shortdash_dot shortdash_dot dot dot) lcolor(black gray black gray black gray black gray) ytitle("Cumulative incidence") xtitle("Analysis time (t, days)") graphregion(color(white)) legend(off)

graph save "STCOX_CIF_LTF_POA_LEG_NON_COMB_3_BG", replace

*ALL CIFs

gen cif_all_0 = cif_col_0 + cif_leg_0 + cif_ltf_0 + cif_non_0 + cif_poa_0 + cif_unk_0

gen cif_all_1 = cif_col_1 + cif_leg_1 + cif_ltf_1 + cif_non_1 + cif_poa_1 + cif_unk_1

gen cif_all_2 = cif_col_2 + cif_leg_2 + cif_ltf_2 + cif_non_2 + cif_poa_2 + cif_unk_2

gen cif_all_3 = cif_col_3 + cif_leg_3 + cif_ltf_3 + cif_non_3 + cif_poa_3 + cif_unk_3

twoway line cif_all_* _t if _t<=2000, connect(stepstair stepstair stepstair stepstair) sort lcolor(navy orange maroon green) ytitle("Cumulative incidence") xtitle("Analysis time (t, days)") graphregion(color(white)) legend(off)graphregion(color(white)) legend(off)

graph save "STCOX_CIF_ALL", replace

******************************************************************************

******************************************************************************

******************************************************************************

******************************************************************************

******************************************************************************

******************************************************************************

****USING X-LTF DATASET (RECOVERED LTF) FOR CHECKS*********************

**Reclassifying LTF known fates

use "Supp_Dataset.dta", replace

tab collar_status2 cause_death2

list wolf_ID date_endpoint last_contact collar_recovery_death cause_death cause_endpoint if collar_status2==2 & cause_death2!=.

replace cause_endpoint = "x-ltf" if collar_status2==2 & cause_death2!=.

tab cause_endpoint

save "Supp_Dataset_xLTF.dta", replace

list wolf_ID date_endpoint cause_endpoint if year_endpoint<=1984 & month_endpoint>=4 & month_endpoint<=9

rename cause_endpoint2 old_endpoint

tab old_endpoint cause_endpoint

encode cause_endpoint, gen(cause_endpoint_enc)

label var cause_endpoint_enc "coded endpoint"

order cause_endpoint_enc, after(cause_endpoint)

tab cause_endpoint cause_endpoint_enc

expand 7

by wolf_ID, sort: gen cause_endpoint2= _n+1

order cause_endpoint2, after(cause_endpoint_enc)

tab cause_endpoint2 cause_endpoint_enc

/*Generating cause of endpoint binary variables*/

gen collision = cause_endpoint2==2

gen legal = cause_endpoint2==3

gen ltf = cause_endpoint2==4

gen nonhuman = cause_endpoint2==5

gen poached = cause_endpoint2==6

gen unknown = cause_endpoint2==7

gen x_ltf = cause_endpoint2==8

gen event = (cause_endpoint2==cause_endpoint_enc)

gen censored = cause_endpoint_enc==1

*Generate ID var for each expanded record (wolf_ID - [1-6]) (CLUSTER FOR THIS INSTEAD OF wolf_ID)

gen wolf_ID_exp=wolf_ID+"-"+string(cause_endpoint2, "%02.0f")

order wolf_ID_exp, after(wolf_ID)

*Checking stset

stset date_endpoint, failure(event) exit(failure) origin(time capture_date) id(wolf_ID_exp)

*****TIME-SPLITTING for time-dependent variables and 'spells'*****

stsplit year_split, at(7129 7258 7310 7410 7495 7624 7674 7775 7860 7989 8038 8140 8225 8354 8402 8505 8590 8719 8773 8871 8956 9085 9137 9236 9321 9450 9501 9601 9686 9815 9865 9966 10051 10180 10229 10332 10417 10546 10600 10697 10782 10911 10964 11062 11147 11276 11328 11427 11503 11641 11692 11793 11869 12007 12056 12158 12234 12372 12420 12523 12599 12737 12791 12888 12964 13102 13155 13254 13330 13468 13519 13619 13695 13833 13883 13984 14060 14198 14247 14349 14425 14563 14611 14715 14791 14929 14982 15080 15156 15294 15346 15445 15521 15659 15710 15810 15886 16024 16074 16176 16252 16390 16438 16541 16617 16755 16809 16906 16982 17120 17173 17271 17347 17485 17537 17637 17713 17851 17901 18002 18078 18216 18265 18367 18443 18581 18629 18732 18808 18946 18992 19095 19098) after(capture_date==1/1/1960)

**Generating intra-year periods time-dep binary variable

gen risk_season = 0

label var risk_season "intra-year periods"

replace risk_season = 1 if year_split==7129 | year_split==7495 | year_split==7860 | year_split==8225 | year_split==8590 | year_split==8956 | year_split==9321 | year_split==9686 | year_split==10051 | year_split==10417 | year_split==10782 | year_split==11147 | year_split==11503 | year_split==11869 | year_split==12234 | year_split==12599 | year_split==12964 | year_split==13330 | year_split==13695 | year_split==14060 | year_split==14425 | year_split==14791 | year_split==15156 | year_split==15521 | year_split==15886 | year_split==16252 | year_split==16617 | year_split==16982 | year_split==17347 | year_split==17713 | year_split==18078 | year_split==18443 | year_split==18808 | year_split==19098

replace risk_season = 2 if year_split==7258 | year_split==7624 | year_split==7989 | year_split==8354 | year_split==8719 | year_split==9085 | year_split==9450 | year_split==9815 | year_split==10180 | year_split==10546 | year_split==10911 | year_split==11276 | year_split==11641 | year_split==12007 | year_split==12372 | year_split==12737 | year_split==13102 | year_split==13468 | year_split==13833 | year_split==14198 | year_split==14563 | year_split==14929 | year_split==15294 | year_split==15659 | year_split==16024 | year_split==16390 | year_split==16755 | year_split==17120 | year_split==17485 | year_split==17851 | year_split==18216 | year_split==18581 | year_split==18946

replace risk_season = 3 if year_split==6946 | year_split==7310 | year_split==7674 | year_split==8038 | year_split==8402 | year_split==8773 | year_split==9137 | year_split==9501 | year_split==9865 | year_split==10229 | year_split==10600 | year_split==10964 | year_split==11328 | year_split==11692 | year_split==12056 | year_split==12420 | year_split==12791 | year_split==13155 | year_split==13519 | year_split==13883 | year_split==14247 | year_split==14611 | year_split==14982 | year_split==15346 | year_split==15710 | year_split==16074 | year_split==16438 | year_split==16809 | year_split==17173 | year_split==17537 | year_split==17901 | year_split==18265 | year_split==18629 | year_split==18992

label define risk_seasonl 0 "NONE" 1 "hunt/hound" 2 "hunt/hound/snow" 3 "snow"

label values risk_season risk_seasonl

tab risk_season

tab risk_season if _d==1

tab cause_endpoint_enc risk_season if _d==1

*****PROTECTION PERIOD VARIABLE*****

stsplit treat_split, at(15795 16466 16526 16692 16914 17013 17236 17803 18020 18078 19018 19097) after(capture_date==1/1/1960)

***Generating liberalized killing treatment binary variable (1 if lib kill period)

gen lib_kill = 0

replace lib_kill = 1 if treat_split==15795 | treat_split==16526 | treat_split==16914 | treat_split==17236 | treat_split==18020 | treat_split==19018

order lib_kill treat_split, after(cause_endpoint_agg)

tab lib_kill if _d==1

/*346 deaths outside lib kill periods; 109 within*/

tab cause_endpoint2 lib_kill if _d==1

/*very few (<10) observations for collision (3) & unknown (7) and x-ltf (6) within lib kill periods*/

tab risk_season lib_kill if _d==1

*censoring all other created spells

replace event=0 if event==.

***SAVE DATASET***

save "Supp_Dataset_expanded_xLTF.dta", replace

tab cause_endpoint_enc if _d==1

******************************************************************************

*JOINT ST COX MODELS FOR ALL CAUSE-SPECIFIC HAZARDS********************

******************************************************************************

*Checking stset by wolf_ID_exp (for multiple records)

stset date_endpoint, failure(event) exit(failure) origin(time capture_date) id(wolf_ID_exp)

stdes

stsum

******************************************************************************

***CAUSE-SPECIFIC HAZARD RATES FOR ALL CAUSES SIMULTANEOUSLY*******

*Fit all in same model (basically same results as with separate PH models)

stcox 1.risk_season#2.cause_endpoint2 1.risk_season#3.cause_endpoint2 1.risk_season#4.cause_endpoint2 1.risk_season#5.cause_endpoint2 1.risk_season#6.cause_endpoint2 1.risk_season#7.cause_endpoint2 1.risk_season#8.cause_endpoint2 2.risk_season#2.cause_endpoint2 2.risk_season#3.cause_endpoint2 2.risk_season#4.cause_endpoint2 2.risk_season#5.cause_endpoint2 2.risk_season#6.cause_endpoint2 2.risk_season#7.cause_endpoint2 2.risk_season#8.cause_endpoint2 3.risk_season#2.cause_endpoint2 3.risk_season#3.cause_endpoint2 3.risk_season#4.cause_endpoint2 3.risk_season#5.cause_endpoint2 3.risk_season#6.cause_endpoint2 3.risk_season#7.cause_endpoint2 3.risk_season#8.cause_endpoint2, efron strata(cause_endpoint2 lib_kill) robust cluster(wolf_ID)

estimates store fullperiod_KFstrat

estat ic

/*Sample code for checking assumptions*/

estat phtest, log detail

estat phtest, plot(1.risk_season#4.cause_endpoint2)

estat phtest, plot(2.risk_season#4.cause_endpoint2)

estat phtest, plot(1.risk_season#5.cause_endpoint2)

estat phtest, plot(2.risk_season#5.cause_endpoint2)

estat phtest, plot(3.risk_season#5.cause_endpoint2)

estat phtest, plot(2.risk_season#7.cause_endpoint2)

stphplot if cause_endpoint2==2, by(risk_season) nolnt

stphplot if cause_endpoint2==3, by(risk_season) nolnt

stphplot if cause_endpoint2==4, by(risk_season) nolnt

stphplot if cause_endpoint2==5, by(risk_season) nolnt

stphplot if cause_endpoint2==6, by(risk_season) nolnt

stphplot if cause_endpoint2==7, by(risk_season) nolnt

stphplot if cause_endpoint2==8, by(risk_season) nolnt

*WITH TVCs

stcox 1.risk_season#2.cause_endpoint2 1.risk_season#3.cause_endpoint2 1.risk_season#4.cause_endpoint2 1.risk_season#5.cause_endpoint2 1.risk_season#6.cause_endpoint2 1.risk_season#7.cause_endpoint2 1.risk_season#8.cause_endpoint2 2.risk_season#2.cause_endpoint2 2.risk_season#3.cause_endpoint2 2.risk_season#4.cause_endpoint2 2.risk_season#5.cause_endpoint2 2.risk_season#6.cause_endpoint2 2.risk_season#7.cause_endpoint2 2.risk_season#8.cause_endpoint2 3.risk_season#2.cause_endpoint2 3.risk_season#3.cause_endpoint2 3.risk_season#4.cause_endpoint2 3.risk_season#5.cause_endpoint2 3.risk_season#6.cause_endpoint2 3.risk_season#7.cause_endpoint2 3.risk_season#8.cause_endpoint2, tvc(1.risk_season#5.cause_endpoint2 1.risk_season#8.cause_endpoint2 2.risk_season#5.cause_endpoint2 2.risk_season#7.cause_endpoint2 3.risk_season#5.cause_endpoint2) texp(ln(_t)) efron strata(cause_endpoint2 lib_kill) robust cluster(wolf_ID)

estimates store fullperiod_KFTVCstrat

estat ic

estout fullperiod_KFTVCstrat, eform cells("b(star fmt(3)) ci_l ci_u" se(par fmt(2))) starlevels(* 0.10 ** .05 *** 0.01) stats(ll aic bic, labels("Log likelihood" "AIC" "BIC")) legend
